# Unraveling Puerarin’s impact on MRI hepatic lipid deposition and serum lipids in IUGR offspring rats

**DOI:** 10.1371/journal.pone.0350859

**Published:** 2026-06-12

**Authors:** Kalonda Alpha Mutamba, DuJun Bian, Tao Wang, Xiao Ri He

**Affiliations:** 1 Department of Pediatrics, The Second Xiangya Hospital, Central South University, Changsha, Hunan, China; 2 Department of Radiology, The Second Xiangya Hospital, Central South University, Changsha, Hunan, China; 3 Department of Neonatology, Children’s Medical Center, The Second Xiangya Hospital, Central South University, Changsha, Hunan, China; University of Life Sciences in Lublin, POLAND

## Abstract

Intrauterine growth restriction (IUGR) is associated with long-term metabolic programming effects, including hepatic structural alterations and later-life susceptibility to metabolic disease, potentially involving dysregulation of peroxisome proliferator-activated receptor alpha (PPARα), a key regulator of hepatic fatty acid oxidation. Given its established antioxidant and lipid-modulating properties in adult models of metabolic syndrome, puerarin, a natural isoflavone, was evaluated as an early-life intervention to mitigate hepatic injury in IUGR offspring. An IUGR rat model was established via gestational protein restriction. Male offspring were randomized into Control, IUGR, and IUGR plus puerarin groups. Separate cohorts (n = 6 per group per time point) were assessed at weeks 3, 8, and 12. Puerarin (50 mg/kg/day) was administered intraperitoneally from postnatal days 7–21. Hepatic tissue characteristics were evaluated using T1 mapping and intravoxel incoherent motion (IVIM) MRI. Serum lipid profiles and hepatic PPARα mRNA expression were also measured. Data were analyzed using two-way ANOVA followed by Tukey’s post hoc test. IUGR offspring exhibited significantly elevated hepatic T1 relaxation times from week 3 onward (*P* < 0.0001), indicating early and persistent hepatic microstructural alterations. Serum lipid parameters remained largely comparable across all groups, with no significant intergroup differences in TG, TC, LDL-C, or HDL-C at individual time points. Puerarin treatment significantly reduced T1 values and improved diffusion-related parameter (D) across all time points (*P* < 0.05), indicating improved hepatic water mobility and microstructural integrity. ADC showed early improvement at week 3, whereas perfusion-related IVIM parameters (D* and F) showed no consistent or significant intergroup differences. Serum TG levels were significantly reduced by puerarin at week 12 compared with untreated IUGR offspring (*P* < 0.05), while TC and LDL-C remained unchanged. Hepatic PPARα expression was markedly suppressed in IUGR offspring across all time points (*P* < 0.0001) and was significantly increased by puerarin only at week 12 (*P* < 0.01), although overall expression remained below control levels. These findings suggest that IUGR primarily induces early hepatic microstructural alterations detectable by quantitative MRI before overt systemic dyslipidemia. Early-life puerarin intervention partially ameliorates hepatic diffusion abnormalities and improves selected metabolic markers, potentially through delayed activation of lipid oxidation pathways including PPARα. However, further studies incorporating direct hepatic lipid quantification and functional metabolic assessments are required to validate these findings.

## Introduction

Intrauterine Growth Restriction (IUGR), defined as impaired fetal growth resulting in a birth weight below the 10^th^ percentile or two standard deviations below gestational norms, is a major contributor to perinatal complications and chronic long-term health issues [1]. As a leading cause of perinatal mortality and morbidity, IUGR affects approximately 7–15% of pregnancies worldwide [2]. In China, the reported incidence is 8.77%, affecting roughly 1.6 million newborns annually and placing a significant burden on public health [3]. The pathogenesis of IUGR is multifactorial, with risk factors including maternal pregestational diabetes mellitus, fetal chromosomal abnormalities such as trisomy 13, and placental insufficiency [4].

Individuals born with IUGR are particularly predisposed to developing non-alcoholic fatty liver disease (NAFLD) due to adaptive responses to nutrient deprivation during critical developmental windows [5]. Notably, the prevalence of NAFLD in this population is up to four times higher than in children born at a normal weight [6]. Mechanistic studies suggest that fetal nutrient deprivation leads to maladaptive programming of hepatic lipid metabolism [7]. Furthermore, the catch-up growth represents a critical developmental window where the mismatch between a restricted intrauterine environment and a calorie-sufficient postnatal environment accelerates the development of NAFLD and hyperlipidemia [8,9].

Central to this regulation are peroxisome proliferator-activated receptors (PPARs), nuclear transcription factors that govern lipid storage and oxidation. While Peroxisome Proliferator-Activated Receptor gamma (PPARγ) promotes adipocyte differentiation and lipid accumulation [10], PPARα is highly expressed in the liver and governs fatty acid β-oxidation and energy homeostasis [11]. Previous research has implicated reduced hepatic PPARα expression in the impaired fatty acid clearance and subsequent lipid accumulation observed in IUGR offspring [10].

Identifying postnatal interventions to restore these metabolic pathways is therefore a clinical priority. Puerarin, a bioactive isoflavone derived from the kudzu root (Pueraria lobata), possesses multifaceted effects on lipid metabolism and mitochondrial function that may counteract the metabolic sequelae of IUGR. Prior studies demonstrate that puerarin reduces serum triglycerides (TG) and total cholesterol (TC), suppresses hepatic lipogenesis, and enhances β-oxidation enzymes via PPARα activation in adult rodent models [12,13]. Puerarin exerts anti-lipogenic effects by activating adenosine monophosphate-activated kinase (AMPK) and suppressing key regulators such as sterol regulatory element-binding protein 1c (SREBP-1c), fatty acid synthase (FAS), and acetyl-CoA carboxylase (ACC) [14–17]**.** Concurrently, it enhances mitochondrial integrity through the upregulation of PPARα and associated enzymes, including carnitine palmitoyltransferase-1 (CPT-1), and acyl-CoA oxidase (ACOX) in liver and skeletal muscle [17–19]**.** It also stimulates cholesterol efflux via the AMPK-PPARγ-LXR-ABCA1 axis, which is beneficial for intracellular lipid resolution [20]. Together, these mechanisms provide a strong rationale for investigating whether early-life puerarin administration can mitigate IUGR-related hepatic disturbances by restoring PPARα-dependent lipid oxidation.

To test this hypothesis, we employed advanced quantitative magnetic resonance imaging (QMRI) techniques, including T1 mapping [21], and intravoxel incoherent motion (IVIM) imaging [22], alongside biochemical assays to comprehensively assess the effects of postnatal puerarin on hepatic tissue composition, microvascular perfusion, serum lipid profiles, and PPARα expression in the IUGR rat model. Understanding these interactions is a necessary step toward developing targeted strategies for managing the long-term hepatic and metabolic complications associated with IUGR.

## Materials and methods

Specific-pathogen-free Sprague-Dawley rats (n = 40; 20 male, 20 female; 180−220 g; 9 weeks old) were housed under controlled conditions (22 ± 2°C; 50%−80% humidity; 12-hour light/dark cycle) following ethical approval from the Institutional Animal Care and Use Committee (CSU-2023–0244). Rats were acclimated for one week with ad libitum access to a standard chow diet and water before pairing (1:1). Pregnancy was confirmed by sperm-positive vaginal smear (gestational day 0).

Pregnant dams were randomized into two groups (n = 10 per group): Control (fed 21% protein diet) and IUGR (fed 10% protein diet). Offspring were weighed within 24 hours of birth, and IUGR status was defined as a birth weight below the mean minus two standard deviations of the control group, consistent with established rodent IUGR models [23]. To minimize sex-related variability in lipid metabolism, only male pups were included in the postnatal experimental groups. Male offspring were allocated into three groups: Control, IUGR, and IUGR plus puerarin (n = 6 per group). The sample size was determined based on previous studies investigating IUGR and metabolic outcomes in rats, which demonstrated statistically significant differences with similar group sizes [23–25]. Although a formal power analysis was not performed, the current design aligns with widely used preclinical standards for exploratory studies in IUGR and metabolic research.

The IUGR + puerarin group received intraperitoneal puerarin (50 mg/kg/d) diluted in 5% glucose from postnatal day 7–21 [26,27]. The dosage was selected based on prior studies demonstrating that puerarin at 50 mg/kg exerted significant protective effects on lipid metabolism and hepatic injury [12,23]. Intraperitoneal delivery was chosen to ensure consistent systemic bioavailability in neonatal rats, as oral absorption at this developmental stage is variable. Throughout the 14-day administration, pups were monitored daily for signs of abdominal distress or irritation; no adverse behavioral or physical reactions to the vehicle were observed. Pharmacokinetic studies in rodents have demonstrated that puerarin, when administered systemically, reaches the liver at biologically active concentrations, supporting the plausibility of the hepatic effects observed in the present study [28,29].

At postnatal weeks 3, 8, and 12, separate cohorts of male offspring (n = 6 per group per time point; total N = 54) underwent liver MRI followed by euthanasia for tissue and blood collection. This cross-sectional design was chosen to allow for terminal liver tissue analysis at each developmental stage. A 3.0-T clinical scanner equipped with a small-animal/knee volume coil was used for MRI. T1-weighted images were acquired using a fast spin-echo sequence (e.g., TR/TE = 14.39/7.14ms, FA = 10˚, matrix = 256 × 205, bandwidth = 120HZ/Px, MNS = 2), and T1 maps were analyzed in RadiAnt DICOM Viewer by placing three circular regions of interest (ROIs) of 11–26 mm^2^ within the liver parenchyma, carefully avoiding large vessels and motion artifacts. Diffusion-weighted imaging (DWI) was performed in the axial plane using nine b-values (0, 25, 50, 100, 150, 300, 500, 800, 1000s/mm^2^) for IVIM analysis (TR/TE = 2000/80ms, FA = 90˚, matrix = 256 × 192, MNS = 1), see S1 file for more details. To ensure objectivity, all parameters were extracted using the United Imaging workstation by a researcher blinded to the treatment groups using two 50 mm^2^ ROIs per animal.

Blood samples were collected via cardiac puncture under anesthesia (1% pentobarbital sodium, 50 mg/kg, intraperitoneal). Animals were subsequently euthanized via carbon dioxide (CO_2_) inhalation in a displacement chamber (Yuyan CL-1000M; flow rate 10 L/min), consistent with American Veterinary Medical Association guidelines. All procedures were performed under anesthesia, and every effort was made to minimize animal suffering [30]. Serum was analyzed for TG, TC, high-density lipoprotein cholesterol (HDL-C), and low-density lipoprotein cholesterol (LDL-C) using an automated biochemical analyzer.

Total RNA was extracted from liver tissue (approximately 20 mg) using TRIzol reagent (Invitrogen, USA) according to the manufacturer’s protocol. RNA concentration and purity were determined by absorbance at 260/280 nm. One microgram of total RNA was reverse-transcribed into cDNA using HiFiScript reverse transcriptase (CWBIO, China).

qRT-PCR was performed using SYGR Green Master Mix (Applied Biosystems, USA) on an ABI 7500 system. Each sample was run in triplicate (10 µL per well, 30 µL total reaction volume per sample). The amplification protocol consisted of 95°C for 10 min, followed by 40 cycles of 95°C for 15s and 60°C for 30s, with melting curve analysis from 60–95°C, see S2 file for more details. Primer sequences are shown in Table 1. GAPDH served as the internal control. Relative mRNA expression of PPARα was determined using the 2^- ΔΔ Ct^ method, where △ Ct = Target gene Ct – Internal reference gene Ct, and normalized to the control group.

**Table 1:** Primer sequence of genes used for qRT-PCR.

Gene	Forward Primer (5’-3’)	Reverse Primer (5’-3’)	Product length
**PPARα**	GAATCCACGAAGCCTACC	TAGTCTTTCCTGCGAGTATG	75 bp
**GAPDH**	ACAGCAACAGGGTGGTGGAC	TTTGAGGGTGCAGCGAACTT	252 bp

Data were analyzed using GraphPad Prism (version 8.0.2). The primary effects of treatment, time, and their interaction were assessed using an ordinary two-way ANOVA with Tukey’s post hoc test. Given the small sample size (n = 6), inter-group comparisons at specific time points (week 3,8,12) were performed using the non-parametric Kruskal-Wallis test followed by Dunn’s post hoc test for multiple comparisons. Data are presented as medians and interquartile ranges (IQR). A value of *P* < 0.05 was considered statistically significant.

## Results

### Establishment of the IUGR animal model

**Fig 1:**
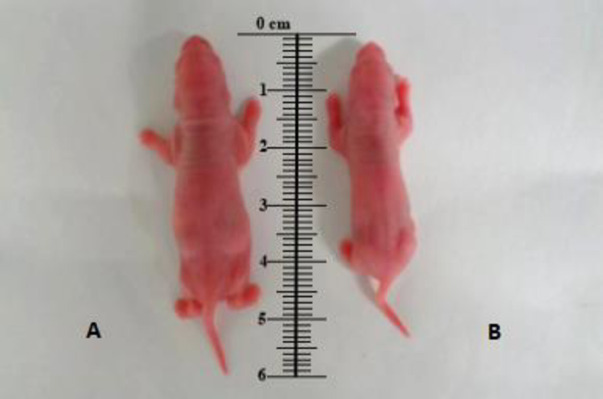
Day 1 Control vs IUGR pup appearance. **(A)** represents the control group pup, and **(B)** represents the IUGR group pup.

The IUGR model was successfully established via a low-protein maternal diet throughout gestation [31]. This dietary manipulation resulted in a significant reduction in birth weight among IUGR offspring (5.23 ± 0.54g) compared with controls (7.36 ± 0.62g; *P* < 0.001), fulfilling the definition of IUGR as birth weight below two standard deviations of the control mean. A representative image comparing control and IUGR pups at postnatal day 1 (Fig 1) highlights the physical growth impairment characteristic of the IUGR phenotype.

### Hepatic T1 mapping and diffusion characteristics

MRI assessments, including T1 mapping and IVIM analysis, were conducted at weeks 3, 8, and 12 across three groups: control, IUGR, and IUGR treated with puerarin.

T1 mapping values were significantly affected by treatment (*F*(2,45)=45.48, *P* < 0.0001), whereas neither the effect of time (*F*(2,45)=1.224, *P* = 0.3036) nor the interaction between time and treatment (*F*(4,45)=1.674, *P* = 0.1727) were statistically significant. Specifically, T1 mapping values were significantly elevated in the IUGR group compared to controls at weeks 3, 8, and 12 (all P < 0.0001), indicating expansion of tissue water content and possible microstructural alteration. In contrast, puerarin treatment significantly reduced T1 mapping values compared to the untreated IUGR group at week 3 (*P = 0.0401*), week 8 (*P < 0.0001*), and week 12 (*P* < 0.0001).

To further characterize the tissue microstructure, water mobility was assessed via the D parameter. Unlike T1 mapping, the D parameter was significantly affected by both treatment (*F*(2,45)=32.29, *P* < 0.0001) and time (*F*(2,45)=74.27, *P* < 0.0001), with a significant interaction between these factors (*F*(4,45)=4.440, *P =* 0.0041). The IUGR group exhibited a significant reduction in D values compared to controls at week 3 (*P* < 0.0001) and week 8 (*P* = 0.0009), suggesting restricted water mobility likely due to increased cellular density. Notably, puerarin treatment significantly increased D values compared to the untreated IUGR group across all time points, respectively, week 3 (*P* < 0.0001), week 8 (P = 0.0082), and week 12 (*P* = 0.0433).

Unlike T1 and D, D* values were not significantly affected by treatment (*F*(2,45)=2.790, *P* = 0.0721) or the interaction between time and treatment (*F*(4,45)=1.303, *P* = 0.2834). However, a significant effect of time was observed (*F*(2,45)=12.30, *P* < 0.001), reflecting physiological changes in perfusion by week 12. There were no significant differences in D* values between the control and IUGR groups at any time point (*P* > 0.05). While puerarin treatment group showed a significant increase in D* values compared with the IUGR group at week 12 (*P* = 0.0219), no significant differences were observed at weeks 3 or 8.

Similar to D*, F values were significantly influenced by time (*F*(2,45)=41.33, *P* < 0.0001) and treatment (*F*(2,45)=3.808, *P* < 0.0297), with no significant interaction (*P* = 0.509). However, the F values did not differ significantly between IUGR and control groups at any specific week (*P* > 0.05). Furthermore, puerarin treatment did not significantly alter the perfusion fraction compared to IUGR group across the 12-week period (*P* > 0.05).

**Fig 2:**
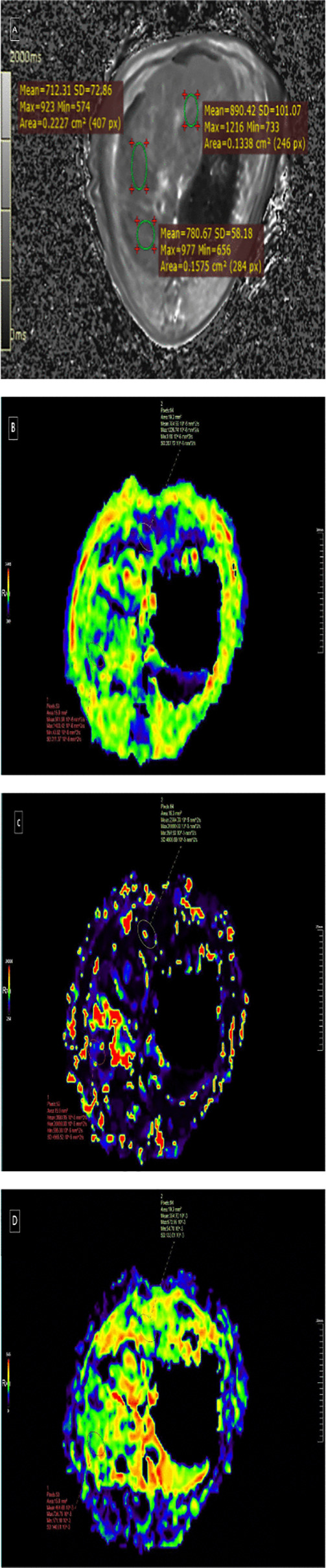
Hepatic T1 mapping and IVIM MRI. **(A)** T1 mapping, **(B)** IVIM slow diffusion coefficient D, **(C)** IVIM pseudo-diffusion D*, and **(D)** IVIM perfusion fraction F. The images show week 8 control group hepatic MRI.

**Fig 3:**
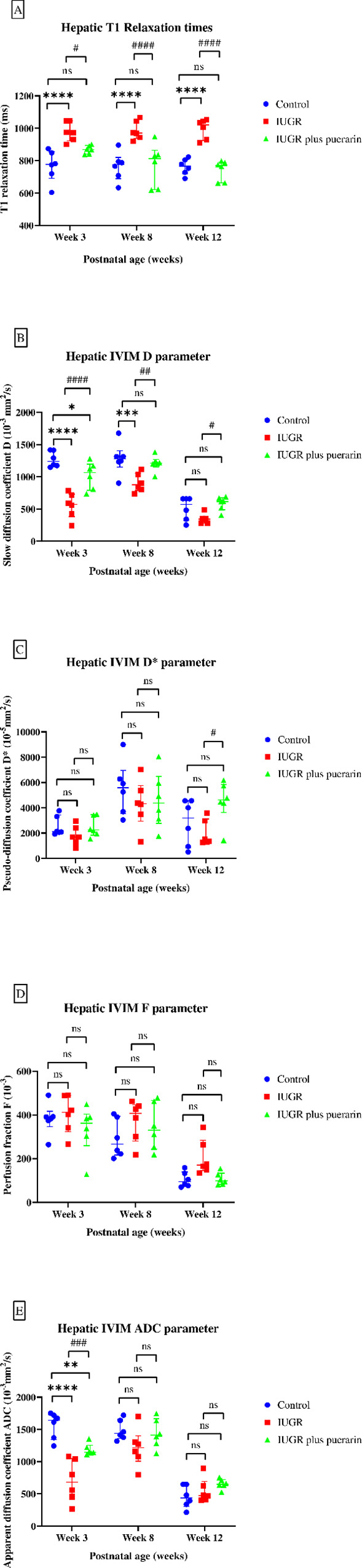
Quantitative analysis of hepatic T1 relaxation times and IVIM-derived parameters. **(A)** Hepatic T1 relaxation times, **(B)** Slow diffusion coefficient D, **(C)** Pseudo-diffusion coefficient D*, **(D)** Perfusion fraction F, and **(E)** Apparent diffusion coefficient ADC. Individual data points (n = 6 per group) are presented as scatter plots, with horizontal lines representing the Mean ± SD. Overall effects of treatment, time, and their interaction were assessed via ordinary two-way ANOVA. Specific inter-group significance at each timepoint was determined by Tukey’s multiple comparisons test. Week 3 represents the immediate conclusion of the 14-day puerarin treatment window (postnatal days 7-21); inter-group differences at this stage reflect incipient recovery, while longitudinal trends are observed through weeks 8 and 12. ****P < 0.0001, ***P < 0.001, **P < 0.01, *P < 0.05 vs. Control; ^####^*P* < 0.0001, ^###^*P* < 0.001, ^##^*P* < 0.01, ^#^*P* < 0.05 vs. IUGR; ns, non-significant (*P* > 0.05).

ADC values were significantly impacted by treatment (*F*(2,45)=13.56, *P* < 0.0001), time (*F*(2,45)=75.09, *P* < 0.0001), and their interaction (*F*(4,45)=8.016, *P* < 0.0001). At week 3, the IUGR group exhibited a significant reduction in ADC compared to controls (*P* < 0.0001), which was significantly reversed by puerarin treatment (*P* = 0.0007). By weeks 8 and 12, however, no significant differences were observed between the IUGR group and controls (all *P* > 0.05), nor did puerarin treatment result in further significant alterations (See Figs 2 and 3 and Table 2).

**Table 2:** Summary of hepatic MRI parameters.

MRI parameter	Timepoint	Control (n = 6)	IUGR (n = 6)	IUGR plus puerarin (n = 6)
**T1 mapping (ms)**	Week 3	766.6 ± 43.8	978.8 ± 43.8 ****	868.4 ± 43.8 ^#^
	Week 8	763.8 ± 43.8	986.0 ± 43.8 ****	775.2 ± 43.8 ^####^
	Week 12	762.8 ± 43.8	996.4 ± 43.8 ****	741.6 ± 43.8 ^####^
**D (10** ^**–3**^ **mm** ^**2**^ **/s)**	Week 3	1271 ± 95.4	551.1 ± 95.4 ****	1020 ± 95.4 *^, ####^
	Week 8	1280 ± 95.4	906.2 ± 95.4 ***	1206 ± 95.4 ^##^
	Week 12	507.4 ± 95.4	347.6 ± 95.4 ^ns^	584.7 ± 95.4 ^#^
D*(10 ^**–5**^ **mm** ^**2**^ **/s)**	Week 3	2548 ± 902.7	1801 ± 902.7 ^ns^	2481 ± 902.7 ^ns^
	Week 8	5530 ± 902.7	4299 ± 902.7 ^ns^	4604 ± 902.7 ^ns^
	Week 12	2823 ± 902.7	1992 ± 902.7 ^ns^	4489 ± 902.7 ^#^
**F(10**^**−3**^)	Week 3	382.2 ± 47.9	401.7 ± 47.9 ^ns^	331.9 ± 47.9 ^ns^
	Week 8	291.6 ± 47.9	372.6 ± 47.9 ^ns^	345.1 ± 47.9 ^ns^
	Week 12	104.0 ± 47.9	204.9 ± 47.9 ^ns^	106.7 ± 47.9 ^ns^
**ADC (10** ^**–3**^ **mm** ^**2**^ **/s)**	Week 3	1562 ± 121.1	701.8 ± 121.1 ****	1185 ± 121.1 ^###^
	Week 8	1488 ± 121.1	1217 ± 121.1 ^ns^	1436 ± 121.1 ^ns^
	Week 12	452.6 ± 121.1	545.3 ± 121.1 ^ns^	653.9 ± 121.1 ^ns^

Data are presented as Mean ± SD (n = 6 per group). Statistical significance for overall effects was assessed by ordinary two-way ANOVA. Inter-group significance at specific time points was determined using Tukey’s post-hoc multiple comparisons to account for the interaction between time and treatment. ****P < 0.0001, ***P < 0.001, **P < 0.01, *P < 0.05 vs. Control; ^####^*P* < 0.0001, ^###^*P* < 0.001, ^##^*P* < 0.01, ^#^*P* < 0.05 vs. IUGR; ns, non-significant (*P* > 0.05). T1: Longitudinal relaxation time; D: slow diffusion coefficient; D*: Pseudo-diffusion coefficient; F: Perfusion fraction; ADC: Apparent diffusion coefficient.

### Effect of puerarin on serum lipids

Serum TG levels were significantly influenced by both treatment (*F*(2,45)=4.882, *P =* 0.0121) and time (*F*(2,45)=11.68, *P* < 0.0001), while no significant interaction was observed (*P* = 0.5746). TG levels remained statistically comparable between the control and IUGR groups across all time points (all *P* > 0.05). However, at week 12, puerarin treatment resulted in a significant reduction in TG levels compared to the untreated IUGR group (*P* = 0.0186).

Consistent with the TG findings, serum TC levels were also significantly influenced by treatment (*F*(2,45)=4.028, *P =* 0.0246) and time (*F*(2,45)=11.68, *P* < 0.0001), with no interaction effect (*P* = 0.8430). No statistically significant differences were shown between the control, IUGR, and puerarin-treated groups at weeks 3, 8, or 12 (all *P* > 0.05).

Serum HDL levels were significantly influenced by both treatment (*F*(2,45)=4.295, *P* = 0.0197) and time (*F*(2,45)=43.60, *P* < 0.0001), while no significant interaction was observed (*P* = 0.8171). Consistent with the total cholesterol results, HDL levels did not differ significantly between the control, IUGR, and puerarin-treated groups at weeks 3, 8, or 12 (*P* > 0.05).

**Fig 4:**
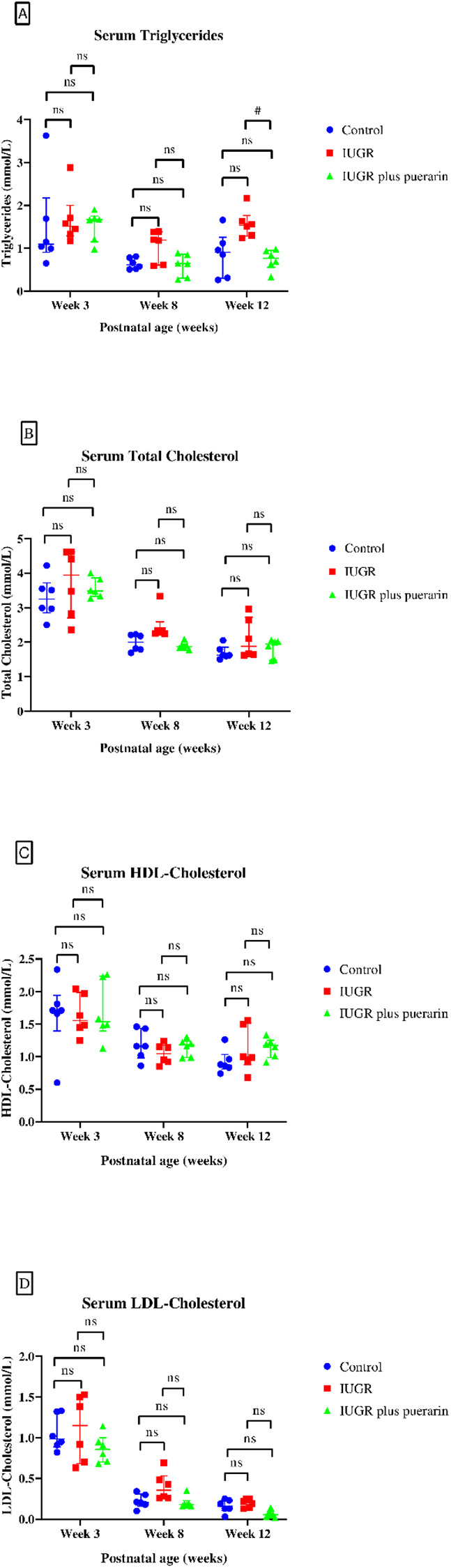
Serum lipid parameters graph. **(A)** Triglycerides (TG), **(B)** Total Cholesterol (TC), **(C)** HDL-Cholesterol, and **(D)** LDL-Cholesterol. Individual data points (n = 6 per group) are presented as scatter plots with horizontal lines representing the Mean ± SD. Overall effects of treatment, time, and their interaction were assessed via ordinary two-way ANOVA. Specific inter-group significance at each timepoint was determined by Tukey’s multiple comparisons test. ^#^*P* < 0.05 vs. IUGR; ns, non-significant (*P* > 0.05).

Similarly, serum LDL levels were significantly influenced by both treatment (*F*(2,45)=5.027, *P* = 0.0107) and time (*F*(2,45)=120.1, *P* < 0.0001), with no significant interaction between the two factors (*P* = 0.727). LDL concentrations revealed no significant differences between control, IUGR, and puerarin-treated groups at weeks 3, 8, and 12 (all *P* > 0.99). See Fig 4 and Table 3.

**Table 3:** Serum lipid parameters.

Serum lipids	Timepoint	Control (n = 6)	IUGR (n = 6)	IUGR plus puerarin (n = 6)
**TG (mmol/L)**	Week 3	1.525 ± 0.29	1.687 ± 0.29 ^ns^	1.520 ± 0.29 ^ns^
	Week 8	0.641 ± 0.29	1.058 ± 0.29 ^ns^	0.601 ± 0.29 ^ns^
	Week 12	0.860 ± 0.29	1.568 ± 0.29 ^ns^	0.735 ± 0.29 ^#^
**TC (mmol/L)**	Week 3	3.290 ± 0.28	3.717 ± 0.28 ^ns^	3.565 ± 0.28 ^ns^
	Week 8	1.987 ± 0.28	2.465 ± 0.28 ^ns^	1.893 ± 0.28 ^ns^
	Week 12	1.698 ± 0.28	2.103 ± 0.28 ^ns^	1.818 ± 0.28 ^ns^
**HDL-C (mmol/L)**	Week 3	1.638 ± 0.18	1.637 ± 0.18 ^ns^	1.695 ± 0.18 ^ns^
	Week 8	1.182 ± 0.18	1.042 ± 0.18 ^ns^	1.142 ± 0.18 ^ns^
	Week 12	0.922 ± 0.18	1.112 ± 0.18 ^ns^	1.137 ± 0.18 ^ns^
**LDL-C (mmol/L)**	Week 3	1.058 ± 0.10	1.110 ± 0.10 ^ns^	0.865 ± 0.10 ^ns^
	Week 8	0.221 ± 0.10	0.400 ± 0.10 ^ns^	0.203 ± 0.10 ^ns^
	Week 12	0.160 ± 0.10	0.196 ± 0.10 ^ns^	0.063 ± 0.10 ^ns^

Data are presented as Mean ± SD (n = 6 per group). Overall treatment effects were assessed by Ordinary two-way ANOVA. Inter-group significance at specific time points was determined using Tukey’s post-hoc multiple comparisons to account for the interaction between time and treatment. ^#^*P* < 0.05 vs. IUGR; ns, non-significant (*P* > 0.05). TG: Triglyceride, TC: Total cholesterol, HDL-C: High-density lipoprotein cholesterol, and LDL-C: Low-density lipoprotein cholesterol.

### Hepatic PPARα mRNA expression

**Fig 5:**
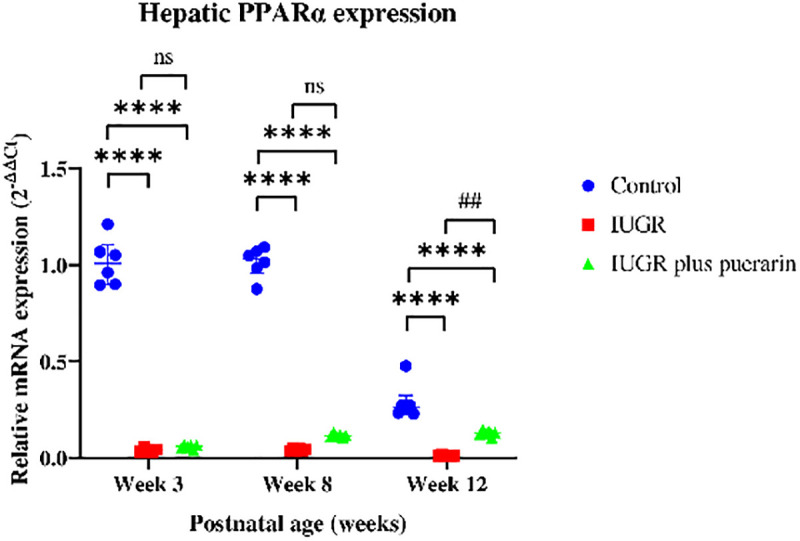
Hepatic mRNA expression of PPARα across study groups. Data are presented as Mean ± SD (n = 6 per group). Overall treatment effects were assessed by Ordinary two-way ANOVA. Inter-group significance at specific time points was determined using Tukey’s post-hoc multiple comparisons to account for the interaction between time and treatment. ***P < 0.001 vs. Control; ns, non-significant (*P* > 0.05).

Hepatic PPARα mRNA expression revealed significant main effects for both treatment (*F*(2,45)=906.3, *P* < 0.0001) and time (*F*(2,45)=102.7, *P <* 0.0001). Furthermore, a significant interaction between treatment and time was observed (*F*(4,45)=102.7, *P <* 0.0001). Specifically, Hepatic PPARα mRNA expression was significantly suppressed in the IUGR group compared to controls across all time points (*P* < 0.0001). While puerarin treatment showed no significant effect at weeks 3 and 8, a significant increase in PPARα expression was observed in the IUGR plus puerarin group compared to the untreated IUGR group by week 12 (*P* = 0.0032). See Fig 5 and Table 4.

**Table 4:** Hepatic mRNA expression of PPARα across study groups.

Timepoint	Control (n = 6)	IUGR (n = 6)	IUGR plus puerarin (n = 6)
**Week 3**	1.014 ± 0.03	0.040 ± 0.03 ^****^	0.058 ± 0.03 ^****^
**Week 8**	1.014 ± 0.03	0.043 ± 0.03 ^****^	0.116 ± 0.03 ^****^
**Week 12**	0.290 ± 0.03	0.011 ± 0.03 ^****^	0.127 ± 0.03 ^****, ##^

Data are presented as Mean ± SD (n = 6 per group). Overall treatment effects were assessed by Ordinary two-way ANOVA. Inter-group significance at specific time points was determined using Tukey’s post-hoc multiple comparisons to account for the interaction between time and treatment. ***P < 0.001 vs. Control; ns, non-significant (*P* > 0.05).

## Discussion

### Hepatic microstructure and perfusion dynamics: MRI insights

Quantitative MRI provided non-invasive, longitudinal insights into hepatic alterations in IUGR offspring. T1 mapping reflects changes in the extracellular matrix, fibrosis, and lipid deposition [32–37]. In our model, IUGR offspring exhibited significantly elevated hepatic T1 values from week 3 through week 12 (*P* < 0.0001), suggesting a persistent expansion of the extracellular space. While some studies suggest that high fat content can reduce T1 values [37,38], an elevated T1 is a hallmark of increased extracellular space, typically seen in edema or early-stage fibrosis [32,39]. In our study, a significant reduction of T1 values following puerarin treatment begun at week 3(*P* = 0.0401) and reached high significance by weeks 8 and 12 (*P* < 0.0001). Recent research confirms that T1 mapping is highly sensitive to early parenchymal changes in pediatric metabolic models, even before histological changes are evident [40,41]. The existence of fat can confound T1 measurements [42,43], yet the normalization of T1 values in the puerarin-treated group suggests that early intervention may stabilize the hepatic environment during critical growth phases, likely by mitigating inflammation and iron-related influences [44,45].

IVIM-derived parameters further clarified alterations in hepatic microstructure and diffusion characteristics in IUGR offspring. Significant reductions in both D and ADC values were observed in the IUGR group at week 3, indicating early restriction of water mobility and altered tissue cellularity. Reduced ADC values are commonly associated with intracellular lipid accumulation, cellular swelling, and restricted Brownian motion of water molecules in fatty liver disease [46]. Similarly, lower D values may reflect increased cellular density and microstructural disorganization within the hepatic parenchyma. Notably, puerarin treatment significantly increased D values across all time points, suggesting sustained improvement in tissue diffusivity and preservation of hepatic microarchitecture. In contrast, ADC abnormalities appeared transient, as differences between the IUGR and control groups were no longer significant by weeks 8 and 12. This finding suggests that ADC may be more sensitive to early-stage hepatic alterations but less effective in capturing persistent metabolic or structural remodeling during later disease progression. Conversely, the sustained reduction in D values in untreated IUGR offspring indicates that pure molecular diffusion may represent a more stable biomarker of chronic hepatic microstructural injury.

Although D* values showed a significant effect of time, no significant differences were detected between the IUGR and control groups at any individual time point. Interestingly, puerarin treatment increased D* values at week 12 compared with untreated IUGR offspring, suggesting a possible late improvement in microvascular perfusion. This delayed perfusion deficit aligns with the two-fit hypothesis of NAFLD, where fetal programming sets the stage for progressive vascular remodeling [47,48]. Similarly, F values revealed no significant pairwise differences between groups at specific time points. Together, these findings indicate that diffusion abnormalities were more prominent and consistent than perfusion-related changes in IUGR offspring.

Previous studies have reported that reduced diffusion and perfusion parameters in fatty liver disease may reflect hepatocellular ballooning, sinusoidal narrowing, and distortion of hepatic microcirculatory architecture [49,50]. Our findings suggest that puerarin’s primary effect may involve preservation of hepatic microstructure and water diffusivity rather than marked restoration of perfusion metrics. This interpretation is consistent with puerarin’s reported anti-inflammatory and lipid-lowering properties, which may mitigate early cellular injury and tissue disorganization associated with IUGR-related hepatic steatosis [51–53]. Although these imaging biomarkers are frequently associated with hepatic steatosis in previous studies, they cannot directly quantify lipid content in the absence of proton density fat fraction (PDFF) or magnetic resonance spectroscopy. Therefore, they are interpreted as surrogate markers of hepatic tissue remodeling.

### Serum lipid profiles and Puerarin’s metabolic impact

Serum lipid analyses demonstrated systemic metabolic alterations in IUGR offspring. Puerarin treatment significantly reduced serum TG levels at week 12 compared with untreated IUGR offspring, indicating a potential late metabolic benefit. However, puerarin did not significantly alter TC, HDL-C, or LDL-C concentrations at specific time points. These findings suggest that overt circulating dyslipidemia had not fully developed within the 12-week observation period.

The absence of marked serum lipid abnormalities despite alterations in hepatic diffusion and T1 mapping may reflect an early stage of metabolic dysfunction. Previous studies have suggested that fetal growth restriction induces long-term metabolic programming through adaptive mechanisms commonly described as the thrifty phenotype, predisposing offspring to later metabolic disease under nutrient-rich postnatal conditions [54,55]. In the present study, hepatic imaging biomarkers appeared more sensitive than circulating lipid parameters for detecting early IUGR-associated abnormalities, implying that structural and microenvironmental liver changes may precede overt systemic dyslipidemia.

Experimental studies have shown that puerarin can modulate hepatic lipid metabolism through suppression of lipogenesis, enhancement of β-oxidation, and regulation of cholesterol homeostasis pathways [12,56,57]. The significant reduction in TG levels observed at week 12 may therefore indicate partial improvement in hepatic metabolic efficiency. While previous studies in ovariectomized rats or obese models reported increases in HDL-C following puerarin treatment [56,58], our study did not observe significant variations in HDL-C. This suggests that in the specific context of IUGR, puerarin’s primary therapeutic efficacy lies in the reduction of pro-atherogenic apoB-containing lipoproteins rather than the enhancement of reverse cholesterol transport [59,60].

Importantly, this study did not include direct hepatic triglyceride quantification, insulin resistance assessment, or glucose tolerance testing. Therefore, while the imaging findings support the presence of early hepatic alterations in IUGR offspring, the extent of systemic metabolic dysfunction and the mechanistic basis of puerarin’s protective effects require further investigation in future studies.

### Hepatic PPARα expression in IUGR and puerarin effects

PPARα is a ligand-activated nuclear transcription factor that plays a central role in hepatic fatty acid β-oxidation and lipid homeostasis [61–65]. Predominantly expressed in the liver, PPARα regulates multiple genes involved in mitochondrial and peroxisomal fatty acid catabolism, thereby limiting hepatic lipid accumulation and metabolic stress. Consistent with previous studies investigating fetal growth restriction and metabolic programming [11,66,67], the present study demonstrated marked and persistent suppression of hepatic PPARα mRNA expression in IUGR offspring across all time points.

Importantly, puerarin treatment significantly increased hepatic PPARα expression by week 12 compared with untreated IUGR offspring, suggesting a delayed but biologically meaningful restoration of lipid metabolic regulation. This temporal pattern is notable, as the recovery in PPARα expression coincided with improvements in diffusion-related MRI parameters and reduced serum TG levels, supporting the possibility that puerarin progressively ameliorates hepatic metabolic dysfunction through modulation of fatty acid oxidation pathways.

The delayed nature of the PPARα response may indicate that puerarin’s early protective effects are mediated initially through alternative mechanisms before later transcriptional reprogramming occurs. Previous experimental studies have shown that puerarin can regulate hepatic lipid metabolism through multiple interconnected signaling pathways, including AMPK/SREBP-1c signaling, antioxidant activity, and anti-inflammatory effects [18,68]. Therefore, the metabolic and imaging improvements observed in this study are likely multifactorial rather than exclusively dependent on PPARα activation.

Despite the significant restoration of PPARα expression at week 12, most circulating lipid parameters remained comparable between groups, suggesting that hepatic molecular and microstructural alterations may precede overt systemic dyslipidemia in this IUGR model. These findings support the hypothesis that puerarin exerts hepatoprotective effects partly through normalization of hepatic lipid metabolic pathways and preservation of liver microarchitecture.

## Conclusion

In summary, this study demonstrates that IUGR is associated with persistent hepatic microstructural alterations, characterized by elevated T1 relaxation times and impaired diffusion-related MRI parameters. These imaging abnormalities were evident despite the absence of marked circulating dyslipidemia, suggesting that hepatic structural and microenvironmental changes may precede overt systemic metabolic dysfunction in IUGR offspring. Among the IVIM-derived metrics, diffusion-related parameters appeared to be more sensitive and consistent indicators of hepatic injury than perfusion-related measurements.

Early puerarin intervention significantly improved hepatic T1 mapping and diffusion parameters and reduced serum TG levels at week 12, indicating partial preservation of hepatic metabolic and microstructural integrity. Furthermore, puerarin significantly restored hepatic PPARα mRNA expression by week 12, supporting a potential role in improving fatty acid oxidation and hepatic lipid metabolic regulation. The delayed recovery of PPARα expression suggests that puerarin’s hepatoprotective effects may involve both transcriptional regulation and additional pathways. These findings highlight the utility of quantitative MRI for longitudinal assessment of early hepatic alterations in IUGR and suggest that puerarin may represent a promising investigational strategy for mitigating IUGR-associated liver injury. However, these findings remain preliminary and are limited by the absence of direct hepatic triglyceride quantification, histopathological correlation, insulin resistance assessment, and clinical neonatal safety evaluation. Further mechanistic and translational studies are warranted to clarify the long-term therapeutic potential of puerarin in metabolic liver disease associated with fetal growth restriction.

## Supporting information

S1 File MRI parameters. (PDF)

S2 File Quantitative Real-time PCR analysis. (PDF)
