# Supplementary material for: Unraveling Puerarin’s impact on MRI hepatic lipid deposition and serum lipids in IUGR offspring rats
Source: PLoS One. 2026 Jun 12;21(6):e0350859. doi: 10.1371/journal.pone.0350859 (PMC13262855; doi:10.1371/journal.pone.0350859)
Supplement: S1 File — (PDF) [file pone.0350859.s001.pdf]

MRI parameters

The imaging procedure utilized the Shanghai United Imaging uMR790 3.0T MRI system and a specialized 12-channel magnetic resonance coil designed for experimental animals, specifically rats.

T1-weighted imaging was conducted in three imaging planes: coronal, sagittal, and transverse, with specific parameters set for optimal results. These parameters included a Repetition Time (TR) of 14.39 milliseconds (ms), an Echo Time (TE) of 7.14 ms, a Flip Angle (FA) of 10°, a Mean Number of Signals (MNS) of 2, a Matrix Size of 256×205, a Bandwidth (BW) of 120 Hertz per pixel (Hz/Px), and an Acquisition Time of 3 minutes and 12 seconds.

Diffusion-weighted imaging (DWI) was performed in the axial plane, utilizing nine b values (b =0, 25, 50, 100, 150, 300, 500, 800, 1000 s/mm^2) to assess diffusion in different tissues. The parameters for DWI included a TR/TE of 2000/80 ms, an FA of 90°, an MNS of 1, a matrix size of 256×192, and an acquisition time of 5 minutes and 15 seconds.

Variable flip angle T1 mapping was conducted in the axial plane to analyze T1 relaxation times. The parameters for this technique included a TR/TE of 695/10.18 ms, two flip angles (FA 1: 90°, FA 2: 145°), an MNS of 2, a BW of 255 Hz/Px, a matrix size of 320×320, and an acquisition time of 2 minutes and 47 seconds.

For water-suppressed spectra analysis, the Multiple-voxel Point-Resolved Spectroscopy (PRESS) sequence was utilized. The acquisition method involved centering the Volume of Interest within the right hepatic lobe, employing a Volume Saturation Suppression (VSS) pulse obliquely to reduce motion artifacts. The voxel size was initially set at 10×10×15 mm but shimmed to 6×6×7 mm for optimal results. Other parameters included a TR of 2000 ms, a TE of 33 ms, an FA of 90°, a BW of 1000 Hz/Px, 1024 sample points, and an acquisition time of 3 minutes and 15 seconds.

Lastly, unsuppressed water spectra were acquired using the PRESS technique with a Voxel Size of 6×6×7 mm, a TR of 2000 ms, a TE of 33 ms, an FA of 90°, a BW of 1000 Hz/Px, 1024 sample points, and an acquisition time of 3 minutes and 15 seconds. These parameters are essential for characterizing and quantifying different aspects of tissue properties.
