# Supplementary material for: Unraveling Puerarin’s impact on MRI hepatic lipid deposition and serum lipids in IUGR offspring rats
Source: PLoS One. 2026 Jun 12;21(6):e0350859. doi: 10.1371/journal.pone.0350859 (PMC13262855; doi:10.1371/journal.pone.0350859)
Supplement: S2 File — (PDF) [file pone.0350859.s002.pdf]

Quantitative Real-time PCR analysis for mRNA expression

The $2^{-\Delta\Delta Ct}$ method was used to calculate the expression level of our studied genes, where $2^{-\Delta\Delta Ct}$ reflects the ratio of the target gene expression levels of each sample relative to the control sample. △ Ct = Target gene Ct - Internal reference gene Ct, Ct value represents the number of amplification cycles required for the fluorescence signal to reach a detectable threshold in each reaction system. All procedures were conducted in a biological safety cabinet under an aseptic environment.

Step 1: RNA extraction

1. Preparation before the experiment

1 ml of Diethylpyrocarbonate (DEPC) was added to 1L of triple distilled water and heated at 121˚C for 20 minutes. The mixture was then shaken well and left overnight to obtain 0.1% DEPC water. All necessary equipment and consumables for RNA extraction, such as enzyme-free tips and centrifuge tubes, were soaked in 0.1% DEPC water overnight. The next day, all instruments soaked in 0.1% DEPC water were removed, packed in newspaper, sterilized with damp heat at 121˚C for 60 minutes, and dried before use.

2. Trizol extracting total RNA from tissues

(1) Approximately 0.02 g of tissue was preserved in Trizol, and 1 mL of Trizol was added to a homogenizer for thorough grinding. After mixing, the mixture was lysed in the chamber for 5 minutes.

(2) 200 μL of trichloromethane was added to the homogenized mixture, vigorously shaken for 15 seconds, and then allowed to stand at room temperature for 3 minutes.

(3) The mixture was centrifuged at 12000 rpm, 4˚C, for 15 minutes. The upper liquid phase was then taken and transferred to a new RNase-free centrifuge tube.

(4) An equal volume of isopropanol was added to the transferred liquid phase, mixed well, and left to stand at room temperature for 10 minutes.

(5) The mixture was centrifuged at 12000 rpm, 4˚C, for 10 minutes. The supernatant was removed, and 1 mL of 75% ethanol (prepared with sterile DEPC-treated water) was added to wash the precipitate.

(6) Then the mixture was centrifuged at 12000 rpm, 4˚C, for 3 minutes, and the supernatant was removed.

(7) The precipitate was air-dried for 5-10 minutes. Then, 20-30 μL of sterile, enzyme-free

water was added to dissolve and precipitate.

(8) The concentration was measured using a UV spectrophotometer by measuring the absorbance values at 260 nm and 280 nm and calculating the concentration and purity.

Step 2: RNA reverse transcription

Using the total mRNA of the organization as a template, reverse transcription of cDNA was performed.

(1) The reaction system, operational steps, and reverse transcription conditions are detailed in Table 1.

Table 1. Reaction system.

Reagent	20 μL reaction system	Final concentration
**DNTP Mix, 2.5 mM each**	4 μL	500 μM each
**Primer Mix**	2 μL	
**RNA template**	7 μL	50 pg-5 μg
**5×RT Buffer**	4 μL	1×
**DTT, 0.1 M**	2 μL	10 mM
**HiFiScript, 200 U/ μL**	μL	

(2) The reaction mixture was subjected to vortex oscillation mixing, followed by brief centrifugation to collect the solution on the tube wall to the bottom of the tube.

(3) The mixture was incubated at 50˚C for 50 minutes and then at 85˚C for 5 minutes. After the reaction was complete, the mixture was briefly centrifuged and cooled on ice.

(4) The reverse transcripts can be directly used for PCR reactions and fluorescence quantitative PCR reactions. Alternatively, they can be stored at -20˚C for a long time.

Step 3: RT qPCR experiment

1. Experimental method: SYBR method

Primer design: The sequence of the target gene on NCBI was searched for, Primer5 software was used to design primers, and synthesized primers by Beijing Qingke. Specific primer details are provided in Table 2.

Table 2. Primer sequence of genes used for qRT-PCR.

Gene	Forward Primer (5'-3')	Reverse Primer (5'-3')	Product length
**PPARα**	GAATCCACGAAGCCTACC	TAGTCTTTCCTGCGAGTATG	75bp
**GAPDH**	ACAGCAACAGGGTGGTGGAC	TTTGAGGGTGCAGCGAACTT	252bp

(2) System composition: Performed real-time quantitative PCR (3 wells per indicator for each

sample, a total of 30 μL system, with 10 μL per well). The reagents used are specified in Table 3.

Table 3. System composition reagents.

Reagent	Volume
Template (reverse transcription product)	2 μL
Primer R (10 μM)	1 μL
Primer F (10 μM)	1 μL
DdH_2O	11 μL
2X SYBGREEN PCR Master Mix	15 μL

(3) Quantitative PCR amplification program. See Table 4 showing the amplification temperatures and Figure 1 displaying melting curve plots.

Table 4. Amplification temperatures.

Temperature	Time
95˚C	10 minutes
95˚C	15 seconds
60˚C	30 seconds
Analysis of melting curve: 60˚C - 95˚C	

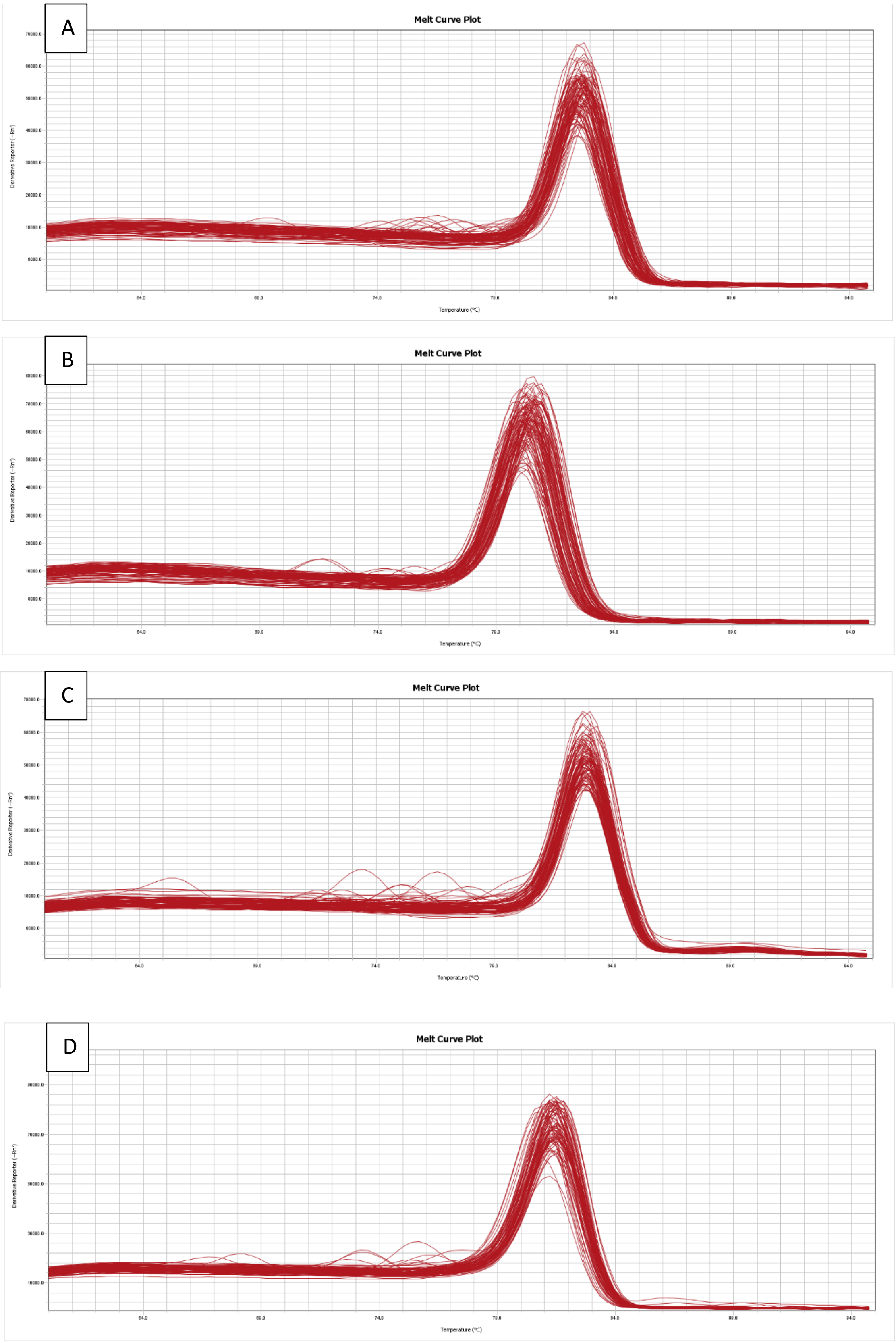
A
Melt Curve Plot
Temperature (°C)
B
Melt Curve Plot
Temperature (°C)
C
Melt Curve Plot
Temperature (°C)
D
Melt Curve Plot
Temperature (°C)

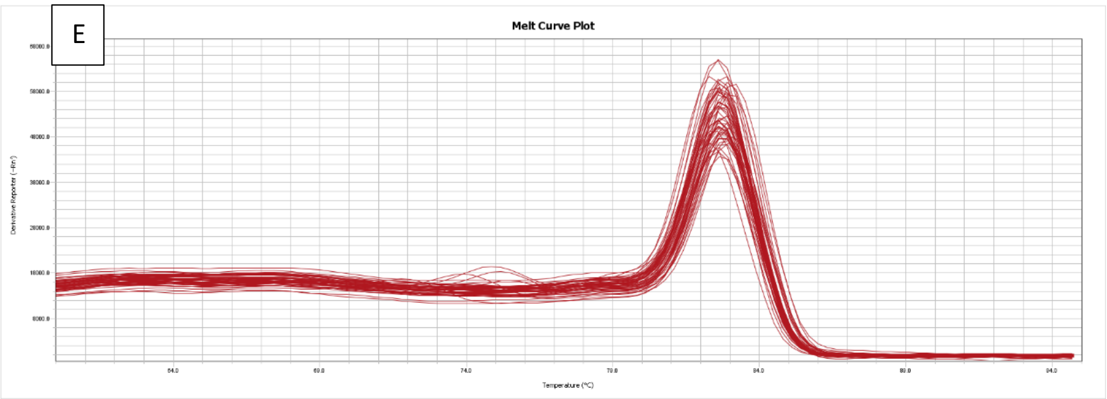

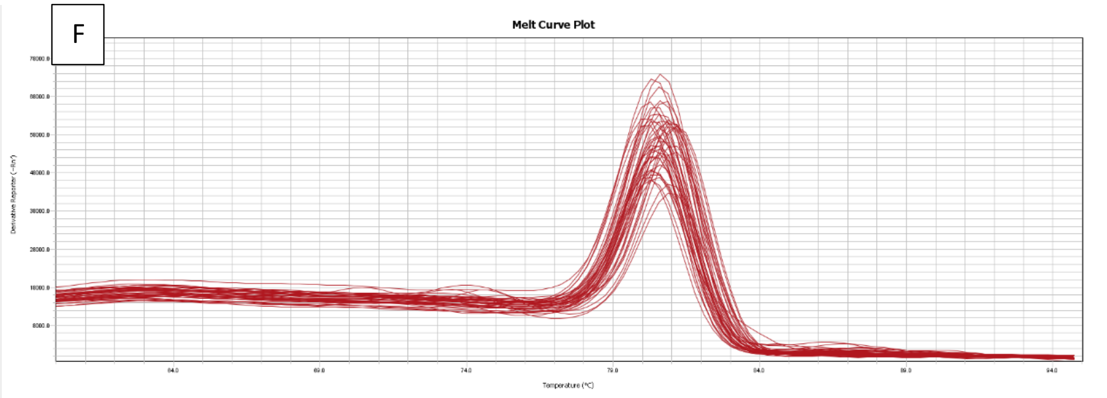

Fig 1. Melting curve plots.

(A) represents the control group PPARα melting curve plot; (B) represents the control group GAPDH melting curve plot; (C) represents the IUGR group PPARα melting curve plot; (D) represents the IUGR group GAPDH melting curve plot; (E) represents the IUGR plus puerarin group PPARα melting curve plot; (F) represents the IUGR plus puerarin group GAPDH melting curve plot.
