# Supplementary material for: Unraveling Puerarin’s impact on MRI hepatic lipid deposition and serum lipids in IUGR offspring rats
Source: PLoS One. 2026 Jun 12;21(6):e0350859. doi: 10.1371/journal.pone.0350859 (PMC13262855; doi:10.1371/journal.pone.0350859)
Supplement: Attachment — Submitted filename: Response to Reviewers.docx [file pone.0350859.s004.docx]

Response to Reviewers

Manuscript title: Unraveling Puerarin’s impact on MRI hepatic lipid deposition and serum lipids in IUGR offspring rats

Manuscript ID: PONE-D-25-33662

Dear Academic Editor and Reviewers,

We thank you very much for your thoughtful and constructive feedback on our manuscript. We have carefully revised the paper in response to your suggestions. Below, we provide a point-by-point response to each comment. All changes are highlighted in the revised manuscript.

Editor

Comment 1:

Please ensure that your manuscript meets PLOS ONE's style requirements, including those for file naming.

Response 1:

We thank the editor for this reminder. We have carefully reviewed the PLOS ONE style requirements and have revised the manuscript and associated files accordingly. File names have been updated to conform to PLOS ONE’s guidelines.

Comment 2:

To comply with PLOS One submissions requirements, in your Methods section, please provide additional information regarding the experiments involving animals and ensure you have included details on methods of anesthesia and/or analgesia, and efforts to alleviate suffering.

Response 2:

We appreciated the editor’s comment. In the revised methods section, we have added detailed information regarding animal welfare, including the methods of anesthesia and euthanasia. Specifically, we now describe that animals were anesthetized with 1% pentobarbital sodium solution (50 mg/kg, intraperitoneal injection) before procedures, and euthanasia was performed using carbon dioxide inhalation following anesthesia. All experiments were conducted in accordance with institutional ethical guidelines and approved protocols.

Comment 3:

Please include this amended Role of Funder statement in your cover letter.

Response 3:

We have added the role of funder statement in our cover letter.

Comment 4:

Please remove any funding-related text from the manuscript and let us know how you would like to update your Funding Statement.

Response 4:

Thank you for pointing this out, we have removed any funding-related text from the acknowledgments section. The funding Statement should be maintained as follows:
Changsha Municipal Bureau of Science and Technology granted the fundings under project number: [kq2007077].

Comment 5:

Please include a separate caption for each figure in your manuscript.

Response 5:

Separate captions have been included for each figure in the manuscript.

Comment 6:

Please include a copy of Table 1, 2, and 3 which you refer to in your text on page 9 and 11.

Response 6:

Thank you very much, all tables referred to have been added to the manuscript.

Comment 7:

[Please include captions for your Supporting Information files at the end of your manuscript, and update any in-text citations to match accordingly. Please see our Supporting Information guidelines for more information: http://journals.plos.org/plosone/s/supporting-information.](http://track.editorialmanager.com/CL0/file:%2F%2F%2FC:%2FUsers%2Fe759388%2FDocuments%2F2025%2FMS%25202025%2FPlease%2520include%2520captions%2520for%2520your%2520Supporting%2520Information%2520files%2520at%2520the%2520end%2520of%2520your%2520manuscript,%2520and%2520update%2520any%2520in-text%2520citations%2520to%2520match%2520accordingly.%2520Please%2520see%2520our%2520Supporting%2520Information%2520guidelines%2520for%2520more%2520information:%2520http:%2Fjournals.plos.org%2Fplosone%2Fs%2Fsupporting-information.%2520/1/010f0198562279b8-ef490006-b8df-4fa3-8c9a-6fdf44f3630e-000000/eb1VQmV659ARXMPHtP2eDpPMqnn9LX-cfDG6GG5IhnI=221)

Response 7:

Thank you very much, this suggestion will be taken into consideration in case of uploading any supporting materials.

Comment 8:

While the introduction effectively outlines the clinical and physiological significance of IUGR, the transition to puerarin as a proposed intervention appears abrupt and insufficiently substantiated. The manuscript would benefit from a clearer mechanistic rationale linking puerarin’s known biological actions such as its effects on peroxisomes, mitochondrial β-oxidation, or SREBP-1c signaling to the specific metabolic disruptions observed in IUGR models.

Response 8:

We have expanded the introduction to provide a clearer mechanistic rationale, incorporating current literature on IUGR pathophysiology, ensuring a smoother and more substantiated transition to puerarin as the proposed intervention. We highlighted that puerarin:

- Suppresses hepatic lipogenesis by activating AMPK and inhibiting SREBP-1c.
- Enhances mitochondrial fatty acid β-oxidation via upregulation of PPARα, thereby improving lipid utilization.
- Promotes cholesterol efflux through the AMPK-PPARγ-LXR-ABCA1 axis.

See page 3, from line 81 of the revised manuscript.

Comment 9:

The experimental design also raises concerns. The number of animals used (n = 6 per group) seems limited for drawing robust conclusions from molecular and physiological data, especially in the absence of a statistical power analysis.

Response 9:

We sincerely thank the editor for raising this important point. We acknowledge that the sample size of six animals per group is modest. The number was chosen based on previous studies using similar IUGR rodent models where n=6 has been considered sufficient to detect meaningful differences in liver fat content, serum lipids, and molecular markers.

- In a rat IUGR model examining hepatic cholesterol metabolism, n=6 per group was employed to detect differences in cholesterol, LDL receptor levels, and related proteins. [Zinkhan, E., Chin, J., Zalla, J. *et al.* Combination of intrauterine growth restriction and a high-fat diet impairs cholesterol elimination in rats. *Pediatr Res* **76**, 432–440 (2014)]
- A research into curcumin’s effects on insulin resistance and lipid accumulation in IUGR rats also used n=6 per group, yielding meaningful outcomes across metabolic parameters. [Niu Y, He J, Ahmad H, Wang C, Zhong X, Zhang L, et al. Curcumin attenuates insulin resistance and hepatic lipid accumulation in a rat model of intra-uterine growth restriction through insulin signalling pathway and sterol regulatory element binding proteins. Br J Nutr. 2019;122(6):616-24]

The related literatures have been referenced in the materials and methods section found on page 3, from line 115.

Comment 10:

Additionally, the criteria used to define IUGR status are not clearly stated (e.g., birth weight <2 SD), and it is unclear whether sex was accounted for in group allocation, despite its known influence on lipid metabolism.

Response 10:

In the revised manuscript materials and methods section, we have clarified that IUGR was defined as offspring birth weight below the mean minus two standard deviations of the control group, consistent with established rodent IUGR models. We also specify that, although both male and female offspring were delivered, only male pups were included in the postnatal experimental groups to minimize sex-related variability in lipid metabolism (Page 3, from line 113).

Comment 11:

Furthermore, the rationale behind the selected puerarin dosage and its route of administration is lacking. The manuscript does not address whether this dosage has been validated in neonatal or IUGR models, nor whether it achieves bioactive concentrations in liver tissue. The absence of pharmacokinetic context weakens the interpretation of the intervention’s biological plausibility.

Response 11:

We thank the reviewer for raising this important point. In the revised materials and methods section, we have clarified the rationale for both puerarin dosage and the route of administration. The dosage of 50mg/kg/day was selected based on previous rodent studies in neonatal and metabolic disease models, where puerarin at this level exerted significant protective effects on hepatic injury and lipid metabolism. We chose the intraperitoneal route to ensure consistent systemic exposure in neonatal rats, since oral absorption can be variable at this stage of development. Furthermore, pharmacokinetic and tissue distribution studies have demonstrated that systemically administered puerarin reaches the liver at biologically active concentrations. These clarifications have now been incorporated into the revised manuscript (Page 4, from line 120)

Comment 12:

Importantly, although the study presents gene and protein expression data related to lipid metabolism (e.g., SREBP-1c, FAS, ACC), there is a notable lack of functional metabolic data. Key physiological parameters such as hepatic triglyceride or cholesterol content, markers of insulin sensitivity, or liver enzyme activity are not reported. As a result, claims regarding "improved lipid metabolism" remain speculative without direct biochemical or phenotypic support. A stronger connection between molecular findings and functional outcomes is necessary to substantiate the conclusions.

Response 12:

We thank the editor for this insightful observation. While we agree that additional functional assays, such as direct hepatic triglyceride/cholesterol quantification, insulin sensitivity testing, or liver enzyme activity, would further strengthen the translational relevance, we would like to emphasize that our study already integrates molecular, biochemical, and imaging assessments.

In addition to examining the expression of lipid metabolism using PPARα, a key regulator of hepatic fatty acid oxidation, we also assessed serum lipid profiles (TG, TC, HDL, LDL) and MRI-derived parameters of hepatic fat and perfusion (T1 mapping, ADC, D, D*, and perfusion fraction F) at multiple time points. These endpoints provide both mechanistic and functional evidence of altered lipid metabolism in IUGR rats and its partial restoration with puerarin treatment.

We have, however, revised the discussion to explicitly acknowledge the absence of direct hepatic biochemical assays and insulin sensitivity markers as a limitation and highlighted that future studies will address these aspects to further validate the molecular and functional findings. (Page 12, line 431)

Comment 13:

In terms of language and style, the manuscript would benefit from a more precise and scientifically rigorous tone. Terms such as "improve lipid metabolism" and "protective effect" are overly general and should be clearly defined and supported by specific outcomes. Several statements in the results section also appear speculative, lacking sufficient data backing (e.g., interpretations of PPARα involvement).

Response 13:

Thank you very much for this suggestion, the manuscript language has been revised and terms have been readjusted to be more precise. And data on PPARα has been indicated in the manuscript.

Comment 14:

Finally, methodological transparency must be improved. The manuscript lacks essential details, such as the complete list of primers used for RT-qPCR, normalization methods for protein expression, and raw numerical data (including means ± SD) underlying the graphs. These omissions hinder reproducibility and limit the reader’s ability to critically assess the findings.

Response 14:

The complete list of of primers used for RT-qPCR, normalization methods for protein expression, and raw numerical data (including means ± SD) underlying the graphs have been added to the manuscript.

Comment 15:

Thank you for uploading this authorship change form. However, the contributions you have indicated for the authors you are requesting to add would not qualify them for authorship. If this was not in error and the individuals in question have not contributed to all of the three points just mentioned but you would like to recognize these individuals for their contribution to the paper, we recommend including them in the Acknowledgments section of your paper.

Response 15:

Thank you very much for guidance on this matter, we have judged well to recognize the individual in the acknowledgments section that has now been written as:

We acknowledge the support of the staff of Central South University's animal facility and laboratory in animal care and experimental procedures.

We also extend our heartfelt gratitude to Doctor Zhou Jun Fang, colleagues and friends who have provided their unwavering assistance for the completion of this research. Be blessed.
