# Supplementary material for: Unraveling Puerarin’s impact on MRI hepatic lipid deposition and serum lipids in IUGR offspring rats
Source: PLoS One. 2026 Jun 12;21(6):e0350859. doi: 10.1371/journal.pone.0350859 (PMC13262855; doi:10.1371/journal.pone.0350859)
Supplement: Attachment — Submitted filename: Response_to_Reviewers_auresp_2.docx [file pone.0350859.s005.docx]

**Response to Reviewers**

Manuscript title: Unraveling Puerarin’s impact on MRI hepatic lipid deposition and serum lipids in IUGR offspring rats

Manuscript ID: PONE-D-25-33662R1

Dear Academic Editor and Reviewers,

We thank you very much for your constructive feedback on our manuscript. We have carefully revised the paper in response to your suggestions. Below, we provide a point-by-point response to each comment, and all changes are highlighted in the revised manuscript.

**Reviewer 2**

**Comment 1:**

Please make sure all the figures should be clear.

Response 1:

We thank the reviewer for this important suggestion. We have revised all figures to ensure clarity.

**Comment 2:**

ANOVA description is incomplete OR incorrect, it state that statistical analysis was performed using one-way ANOVA but the study has 3 groups (Control, IUGR, IUGR + puerarin) and 3 time points (weeks 3, 8, 12). A one-way ANOVA is not appropriate because the design involves two factors: Treatment group and Time Correct approach should be: Two-way ANOVA, or Repeated-measures ANOVA, or Mixed-model ANOVA. Incorrect statistical method reported., please justify or correct it.

Response 2:

We thank the reviewer for this insightful comment and for identifying this error in our statistical reporting. We agree that a one-way ANOVA was not the appropriate method for this experimental design, which involves two independent factors: Treatment Group and Time. We have corrected the 'Statistical Analysis' section of the manuscript to state that a Two-way ANOVA was utilized. This approach allowed us to assess the main effects of the treatment, the effect of time, and the interaction between these two variables. Multiple comparisons were performed using Tukey’s post hoc test to determine significance between groups at individual time points. The revised methodology now accurately reflects the statistical approach used to analyze the study data, and the results have been verified accordingly.

**Comment 3:**

Conflicting MRI results at week 3: significant differences between control and IUGR and IUGR plus puerarin (week 3) but puerarin treatment starts after birth, so: At week 3, the puerarin group should already differ from IUGR (if treatment works). Yet the MRI shows puerarin still identical to IUGR. This contradicts the conclusion that puerarin shows early-life improvement. This is either: A biological inconsistency, or Reporting error please clarify.

Response 3:

We appreciate the reviewer’s rigorous evaluation of the Week 3 data. We would like to clarify that while puerarin administration commenced immediately postnatal, the absence of a significant difference between the IUGR and IUGR plus puerarin groups at the Week 3 MRI timepoint does not indicate a failure of the treatment, but rather reflects the temporal latency required for pharmacological intervention to reverse established intrauterine structural or metabolic programming.

Intrauterine Growth Restriction (IUGR) induces profound developmental adaptations in utero. Although puerarin treatment was initiated shortly after birth, the three-week interval may have been insufficient to manifest detectable changes via MRI, despite the treatment being active. The "early-life improvement" we refer to is characterized by the divergence observed starting between Week 3 and Week 8, where the puerarin group begins to shift toward the Control profile while the untreated IUGR group progresses toward a more severe dyslipidemic state. We have revised the Discussion to more accurately characterize Week 3 as the "initial post-intervention baseline" and clarified that the therapeutic efficacy of puerarin follows a cumulative trajectory that becomes statistically evident by Week 8 and Week 12.

**Comment 4:**

Incomplete reporting of MRI parameters MRI parameters (T1, D, ADC, F, D*) are mentioned, but: Direction of change is never specified (↑ or ↓). No effect sizes or biological interpretation. Only weeks 3, 8, 12 differences are listed. Without describing the direction of abnormalities, the results section is incomplete and unclear.

Response 4:

We thank the reviewer for this constructive critique. We agree that specifying the direction of change and providing a biological interpretation is essential for a complete understanding of the MRI data.

We have revised the Results section to explicitly state the direction of change for each parameter (T1, D, ADC, f, and D*). Furthermore, we have expanded the Discussion to include the biological implications of these findings—specifically how alterations in water diffusion (D, ADC) and perfusion fractions (f, D*) reflect the progressive hepatic or metabolic impairment in the IUGR model and the subsequent restorative effects of puerarin.

**Comment 5:**

The reported IVIM/ADC values in Table 3 are inconsistent with standard units and magnitudes for biological diffusion (values reported as D ≈ 1271 mm²/s and ADC ≈ 1562 mm²/s). Please correct the units and/or scaling (likely ×10⁻³ or ×10⁻⁶) and provide an example calculation or raw output to verify values.

Response 5:

Thank you very much for pointing this out. We have corrected the MRI parameters Table across all time points (Weeks 3, 8, and 12). These corrected magnitudes now align with established physiological ranges for hepatic tissue.

**Comment 6:**

Outdated references used, I recommend adding more recent literature (past 5 years).

Response 6:

We have added more recent references from past 5 years, thank you very much.

**Comment 7:**

Lack of direct hepatic lipid quantification / functional endpoints. The study relies on MRI and serum lipids but does not report direct hepatic triglyceride/cholesterol assays, liver enzymes, or insulin sensitivity measures — the authors acknowledge this but still make claims about “improved lipid metabolism.” Strongly recommend adding if not, require language in the discussion that conclusions are provisional.

Response 7:

Thank you very much for this comment, we have accordingly adjusted the language of the manuscript.

**Comment 8:**

Incorrect or inconsistent reference formatting.

Response 8:

We apologize for the inconsistencies in the reference list. We have conducted a thorough review of the bibliography. Thank you very much.

**Comment 9:**

The manuscript contains numerous grammatical errors, inconsistent terminology and unclear or repetitive sentences that hinder readability. Please check Grammarly.

Response 9:

We sincerely apologize for the linguistic oversights in the original submission. We recognize that clear communication is vital for the dissemination of scientific findings. In response to your feedback, the entire manuscript has undergone a comprehensive revision for clarity, grammar, and style.

**Reviewer 3**

**Comment 1:**

Most previous comments have been addressed, but are not carried through to the abstract, e.g. the abrupt transition to puerarin. The introductory paragraph of the abstract should conclude with the rationale for using puerarin specifically as a therapeutic agent in this study. State the dosage, route of administration, and week of commencement of treatment in the methods component of the abstract.

Response 1:

We thank the reviewer for pointing out these omissions in the abstract. We have fully revised the abstract to ensure consistency with the main text. Specifically: We added a concluding sentence to the introductory section explaining the choice of puerarin based on its known antioxidant and lipid-modulating properties. We now explicitly state the dosage (50 mg/kg/d), the route of administration (intraperitoneal injection), and the treatment window (postnatal days 7 to 21). We have adjusted the transition to puerarin to be more fluid and logically connected to the study's aim of restoring PPARα activity.

**Comment 2:**

‘Whereas puerarin intervention notably reduced serum TG, TC, and LDL levels in IUGR rats.’ Needs to be rephrased – it is not a full sentence.

Response 2:

We apologize for the grammatical error. We have rephrased this statement to ensure it is a complete, grammatically correct sentence that clearly describes the therapeutic effect observed.

**Comment 3:**

Use abbreviations only if a term appears multiple times within the abstract.

Response 3:

We thank the reviewer for this suggestion. We have reviewed the abstract and ensured that all abbreviations are defined at their first usage and subsequently used consistently throughout the text.

**Comment 4:**

Statistical analysis: The standard approach for a study with such a small sample size is to use non-parametric tests (after evaluating the distribution), in particular the Kruskal-Wallis test, using the Conover or Dunn’s test for pairwise comparisons. Please consult a statistician.
Bar graphs of parameters are not ideal because the distribution of the data points is poorly visualized. Box-and-Whisker plots or scatter/dot plots, which are connected to the median and range values shown in the tables, are most likely more suitable. Include the units of measurement in the y-axis title. The images depicted in Fig 2:P Liver T1 mapping and IVIM MRI, sadly, are of poor quality. The script is intelligible and the aspect ratios of the 2nd and 3rd column images are distorted. Please consider improving them.

Response 4:

We thank the reviewer for the suggestion to employ nonparametric testing given our sample size (n=6). We have updated Figures and Tables to present data as Median [Interquartile Range]. Furthermore, inter-group comparisons at each timepoint were recalculated using Kruskal-Wallis tests followed by Dunn’s post-hoc criteria. While this conservative approach slightly adjusted some p-values, the overall longitudinal trends remain robust.

**Comment 5:**

Puerarin administration comment: Puerarin has low water solubility, as you may have noticed. Both puerarin and the 5%D/W solution are irritants and could cause harm and pain when administered intraperitoneally and thereafter due to inflammation. A frequently cited formulation is 10% DMSO + 40% PEG300 + 5% Tween-80 + 45% Saline, as is a 0,9% NaCl solution. (For future reference).

Response 5:

We thank the reviewer for this insightful technical comment regarding the solubility and potential irritant properties of the puerarin formulation. We acknowledge that puerarin's low water solubility often necessitates specialized vehicles like DMSO or PEG for optimal delivery. In this study, we utilized a 5% glucose (D/W) solution as a vehicle based on prior established protocols for neonatal rat models, aiming to minimize the use of organic solvents like DMSO, which can sometimes exert independent metabolic or neuroprotective effects in developing pups. Throughout the 14-day administration period (P7–P21), all pups were closely monitored for signs of abdominal tenderness, lethargy, or behavioral changes. No adverse reactions or significant signs of distress were observed. During the scheduled sacrifices at weeks 3, 8, and 12, the peritoneal cavity and liver surface were inspected; no gross evidence of peritonitis, adhesions, or chronic inflammation was identified. We appreciate the reviewer’s suggestion of the DMSO/PEG300/Tween-80 formulation and will certainly adopt this more robust vehicle for our future longitudinal studies to ensure optimal solubility and minimize tissue irritation.

**Comment 6:**

Lack of reference to clinical (human) relevance and the feasibility of intervention.

Response 6:

We appreciate the reviewer highlighting the need to contextualize our findings within a clinical framework. We have expanded our Discussion to explicitly address the clinical relevance of this study and the translation of puerarin intervention.

**Comment 7:**

The statement that ‘Early-life interventions that target PPARα-mediated lipid regulation and liver function may represent promising avenues to improve lifelong health’ is rather presumptuous, considering the current status of the research in humans. The current data suggt potential benefits in in vitro and animal models, but significant safety concerns, particularly regarding development, and a complete absence of human neonatal clinical trial data mean it is not an approved or recommended treatment for this population. Consider ‘softening’ the statement.

Response 7:

We completely agree with the reviewer’s assessment and thank them for this important correction. Our intention was to highlight the biological potential discovered in our model, but we recognize that the original phrasing overstated the clinical readiness of this intervention. We have "softened" the language throughout the manuscript, particularly in the Abstract and Conclusion. We now explicitly state that these findings are limited to a preclinical rat model and that puerarin is currently neither an approved nor a recommended treatment for human neonates. We have also added a section in the Discussion addressing the significant safety and developmental hurdles that must be cleared before any human neonatal trials could be considered.
