# Supplementary material for: Unraveling Puerarin’s impact on MRI hepatic lipid deposition and serum lipids in IUGR offspring rats
Source: PLoS One. 2026 Jun 12;21(6):e0350859. doi: 10.1371/journal.pone.0350859 (PMC13262855; doi:10.1371/journal.pone.0350859)
Supplement: Attachment — Submitted filename: Response_to_Reviewers_auresp_3.docx [file pone.0350859.s006.docx]

**Response to Reviewers**

Manuscript title: Unraveling Puerarin’s impact on MRI hepatic lipid deposition and serum lipids in IUGR offspring rats

Manuscript ID: PONE-D-25-33662R2

Dear Academic Editor and Reviewers,

We thank you very much for your constructive feedback on our manuscript. We have carefully revised the paper in response to your suggestions. Below, we have provide a point-by-point response to each comment, and all changes have been highlighted in the revised manuscript.

**Reviewer 2**

Comment 1:

MRI data is collected longitudinally (weeks 3, 8, 12), but analyses assume independent observations. If the same animals were followed, appropriate methods (repeated-measures ANOVA or mix-effects models) should be used. Current analysis may yield unreliable results.

Response 1:

We thank the reviewer for this comment and apologize for the lack of clarity regarding our study design. While the data represents different time points (Weeks 3, 8, and 12), these measurements were conducted on separate cohorts of rats at each interval rather than following the same rats. Consequently, the observations are independent, and the use of Two-Way ANOVA is statistically appropriate. We have clarified this in the revised manuscript and updated the figure legends to reflect that each time point represents a distinct group.

Comment 2:

T1 mapping and IVIM diffusion are non-specific and do not directly measure hepatic fat. Conclusions about lipid deposition are not adequately supported without validation (e.g., PDFF, MR spectroscopy, or histology).

Response 2:

We appreciate this important methodological clarification. We acknowledge that T1 mapping and IVIM-derived parameters are indirect, non-specific biomarkers and do not directly quantify hepatic fat content. In the revised manuscript, we have carefully removed all statements implying direct measurement of lipid deposition and replaced them with appropriately cautious terminology referring to hepatic microstructural alterations and water diffusion changes.

Comment 3:

Reported diffusion/ADC values appear far below expected physiological ranges, suggesting possible unit or scaling errors. These should be verified and corrected.

Response 3:

We thank the reviewer for identifying this important issue.In the revised manuscript, we have standardized all diffusion-related parameters and clarified that ADC and D values are reported in units of ×10⁻³ mm²/s.

Comment 4:

The manuscript attributes effects to PPARα activation despite non-significant changes in expression. This represents overinterpretation and should be revised or better supported.

Response 4:

We appreciate this important critique. We acknowledge that our previous interpretation overemphasized the mechanistic role of PPARα. In the revised manuscript, we have tempered all causal statements and clarified that although puerarin significantly increased PPARα expression at week 12, expression remained below control levels. We now interpret PPARα changes as a contributing but not exclusive mechanism, and we have incorporated additional discussion of alternative pathways including AMPK/SREBP-1c signaling and anti-inflammatory effects.

Comment 5:

Effects at week 12 are attributed to early treatment (weeks 1–3) without controlling for confounders (e.g., growth, diet), limiting causal interpretation.

Response 5:

We agree with the reviewer that causal interpretation of long-term effects is limited by potential confounding factors such as growth and developmental progression.We now clearly state that while improvements observed at week 12 coincide with early puerarin administration, definitive causal inference cannot be established within the current study design.

Comment 6:

The manuscript requires language and grammar editing for clarity.

Response 6:

We thank the reviewer for this suggestion. The manuscript has undergone comprehensive language editing and grammatical revision to improve clarity, conciseness, and scientific readability.

Comment 7:

Ensure consistent terminology and definition of abbreviations.

Response 7:

We appreciate this comment.All abbreviations have now been defined upon first use and applied uniformly across text, tables, and figures, including IVIM-derived parameters (D, D*, F), lipid profiles (TG, TC, HDL-C, LDL-C), and PPARα nomenclature.

Comment 8:

Several claims require stronger and more appropriate references, particularly regarding MRI-based lipid assessment.

Response 8:

We thank the reviewer for this valuable suggestion.We have clarified the limitations of MRI in directly quantifying hepatic fat in the absence of PDFF or MR spectroscopy.

Comment 9:

Add methodological details for MRI analysis (e.g., b-values, ROI selection, blinding).

Response 9:

We thank the reviewer for identifying these omissions. We have significantly expanded the MRI Acquisition and Analysis section to include these critical details. Specifically:b-values: We have explicitly listed the nine b-values used for IVIM (0, 25, 50, 100, 150, 300, 500, 800, 1000s/mm^2^). About ROI Selection: We have detailed the size (11–26 mm^2^ for T1 mapping; 50 mm^2^ for IVIM) and the placement (liver parenchyma, avoiding large vessels) of the regions of interest. For blinding: We have clarified that all image post-processing and parameter extraction were performed by a radiologist blinded to the experimental group assignments to ensure objectivity.

Comment 10:

Verify reference formatting and completeness.

Response 10:

Thank you very much for this comment, the reference format has been checked.

**Reviewer 3**

Comment 1:

The proposed analysis is problematic because it switches between parametric (ANOVA) and non-parametric (Kruskal-Wallis) tests for the same data; you should stick to one statistical framework.

Response 1:

We thank the reviewer for this important statistical observation. We agree that using both parametric and non-parametric approaches for the same dataset could create inconsistency in interpretation. In the revised manuscript, we standardized the statistical analysis framework across all continuous variables. Specifically, all outcomes were analyzed using two-way ANOVA (group × time) followed by Tukey’s multiple-comparison post hoc test.
