# Supplementary material for: Unraveling Puerarin’s impact on MRI hepatic lipid deposition and serum lipids in IUGR offspring rats
Source: PLoS One. 2026 Jun 12;21(6):e0350859. doi: 10.1371/journal.pone.0350859 (PMC13262855; doi:10.1371/journal.pone.0350859)
Supplement: Peer review history

# Peer review: Decision Letter 0

**DOI:** 10.1371/journal.pone.0350859.r001

**Authors:** Ewa Tomaszewska

29 Jul 2025

PONE-D-25-33662

Unraveling Puerarin’s impact on MRI hepatic lipid deposition and serum lipids in IUGR offspring rats

PLOS ONE

Dear Dr. Mutamba,

Thank you for submitting your manuscript to PLOS ONE. After careful consideration, we feel that it has merit but does not fully meet PLOS ONE’s publication criteria as it currently stands. Therefore, we invite you to submit a revised version of the manuscript that addresses the points raised during the review process.

Please submit your revised manuscript by Sep 12 2025 11:59PM. If you will need more time than this to complete your revisions, please reply to this message or contact the journal office at plosone@plos.org. When you're ready to submit your revision, log on to https://www.editorialmanager.com/pone/ and select the 'Submissions Needing Revision' folder to locate your manuscript file.

Please include the following items when submitting your revised manuscript:

- A rebuttal letter that responds to each point raised by the academic editor and reviewer(s). You should upload this letter as a separate file labeled 'Response to Reviewers'.
- A marked-up copy of your manuscript that highlights changes made to the original version. You should upload this as a separate file labeled 'Revised Manuscript with Track Changes'.
- An unmarked version of your revised paper without tracked changes. You should upload this as a separate file labeled 'Manuscript'.

If you would like to make changes to your financial disclosure, please include your updated statement in your cover letter. Guidelines for resubmitting your figure files are available below the reviewer comments at the end of this letter.

If applicable, we recommend that you deposit your laboratory protocols in protocols.io to enhance the reproducibility of your results. Protocols.io assigns your protocol its own identifier (DOI) so that it can be cited independently in the future. For instructions see: https://journals.plos.org/plosone/s/submission-guidelines#loc-laboratory-protocols. Additionally, PLOS ONE offers an option for publishing peer-reviewed Lab Protocol articles, which describe protocols hosted on protocols.io. Read more information on sharing protocols at https://plos.org/protocols?utm_medium=editorial-email&utm_source=authorletters&utm_campaign=protocols.

We look forward to receiving your revised manuscript.

Kind regards,

Ewa Tomaszewska, DVM Ph.D

Academic Editor

PLOS ONE

Journal Requirements:

When submitting your revision, we need you to address these additional requirements.

1. Please ensure that your manuscript meets PLOS ONE's style requirements, including those for file naming. The PLOS ONE style templates can be found at https://journals.plos.org/plosone/s/file?id=wjVg/PLOSOne_formatting_sample_main_body.pdf and https://journals.plos.org/plosone/s/file?id=ba62/PLOSOne_formatting_sample_title_authors_affiliations.pdf

2. To comply with PLOS One submissions requirements, in your Methods section, please provide additional information regarding the experiments involving animals and ensure you have included details on methods of anesthesia and/or analgesia, and efforts to alleviate suffering.

3. Thank you for stating the following financial disclosure:

Changsha Municipal Bureau of Science and Technology granted the fundings with project number: [kq2007077]

Please state what role the funders took in the study. If the funders had no role, please state: "The funders had no role in study design, data collection and analysis, decision to publish, or preparation of the manuscript."

If this statement is not correct you must amend it as needed.

Please include this amended Role of Funder statement in your cover letter; we will change the online submission form on your behalf.

4. Thank you for stating the following in the Acknowledgments Section of your manuscript:

We are grateful to the Changsha Municipal Bureau of Science and Technology for granting us funding for this project [kq2007077]. We also acknowledge the support of the staff of Central South University's animal facility and laboratory in animal care and experimental procedures. We also extend our heartfelt gratitude to colleagues and friends who have provided their unwavering assistance for the completion of this research. Be blessed.

We note that you have provided funding information that is not currently declared in your Funding Statement. However, funding information should not appear in the Acknowledgments section or other areas of your manuscript. We will only publish funding information present in the Funding Statement section of the online submission form.

Please remove any funding-related text from the manuscript and let us know how you would like to update your Funding Statement. Currently, your Funding Statement reads as follows:

Changsha Municipal Bureau of Science and Technology granted the fundings with project number: [kq2007077]

Please include your amended statements within your cover letter; we will change the online submission form on your behalf.

5.  Please include a separate caption for each figure in your manuscript.

6. Please include a copy of Table 1, 2, and 3 which you refer to in your text on page 9 and 11.

7. Please include captions for your Supporting Information files at the end of your manuscript, and update any in-text citations to match accordingly. Please see our Supporting Information guidelines for more information: http://journals.plos.org/plosone/s/supporting-information.

8. If the reviewer comments include a recommendation to cite specific previously published works, please review and evaluate these publications to determine whether they are relevant and should be cited. There is no requirement to cite these works unless the editor has indicated otherwise.

Additional Editor Comments :

Dear Authors,

Thank you for submitting your manuscript examining the effects of puerarin on hepatic lipid metabolism in intrauterine growth-restricted (IUGR) piglets. The topic is timely and of potential translational relevance, particularly given the increasing interest in nutritional interventions for metabolic disturbances in early life. However, after careful evaluation, I believe that the manuscript, in its current form, requires substantial revisions before it can be considered for further review.

First, while the introduction effectively outlines the clinical and physiological significance of IUGR, the transition to puerarin as a proposed intervention appears abrupt and insufficiently substantiated. The manuscript would benefit from a clearer mechanistic rationale linking puerarin’s known biological actions such as its effects on peroxisomes, mitochondrial β-oxidation, or SREBP-1c signaling to the specific metabolic disruptions observed in IUGR models. General statements regarding puerarin’s benefits are made, but they are not deeply contextualized with respect to current literature or IUGR pathophysiology.

The experimental design also raises concerns. The number of animals used (n = 6 per group) seems limited for drawing robust conclusions from molecular and physiological data, especially in the absence of a statistical power analysis. Additionally, the criteria used to define IUGR status are not clearly stated (e.g., birth weight <2 SD), and it is unclear whether sex was accounted for in group allocation, despite its known influence on lipid metabolism.

Furthermore, the rationale behind the selected puerarin dosage and its route of administration is lacking. The manuscript does not address whether this dosage has been validated in neonatal or IUGR models, nor whether it achieves bioactive concentrations in liver tissue. The absence of pharmacokinetic context weakens the interpretation of the intervention’s biological plausibility.

Importantly, although the study presents gene and protein expression data related to lipid metabolism (e.g., SREBP-1c, FAS, ACC), there is a notable lack of functional metabolic data. Key physiological parameters such as hepatic triglyceride or cholesterol content, markers of insulin sensitivity, or liver enzyme activity are not reported. As a result, claims regarding "improved lipid metabolism" remain speculative without direct biochemical or phenotypic support. A stronger connection between molecular findings and functional outcomes is necessary to substantiate the conclusions.

In terms of language and style, the manuscript would benefit from a more precise and scientifically rigorous tone. Terms such as "improve lipid metabolism" and "protective effect" are overly general and should be clearly defined and supported by specific outcomes. Several statements in the results section also appear speculative, lacking sufficient data backing (e.g., interpretations of PPARα involvement).

Finally, methodological transparency must be improved. The manuscript lacks essential details, such as the complete list of primers used for RT-qPCR, normalization methods for protein expression, and raw numerical data (including means ± SD) underlying the graphs. These omissions hinder reproducibility and limit the reader’s ability to critically assess the findings.

In summary, while the study addresses an important topic and demonstrates technical merit, significant revisions are necessary to strengthen the experimental rationale, methodological rigor, data interpretation, and clarity of reporting. I encourage the authors to address these issues comprehensively should they choose to revise and resubmit.

Sincerely,

Ewa Tomaszewska

[Note: HTML markup is below. Please do not edit.]

Reviewers' comments:

Reviewer's Responses to Questions

**Comments to the Author**

1. Is the manuscript technically sound, and do the data support the conclusions?

The manuscript must describe a technically sound piece of scientific research with data that supports the conclusions. Experiments must have been conducted rigorously, with appropriate controls, replication, and sample sizes. The conclusions must be drawn appropriately based on the data presented.

Reviewer #1: Yes

**********

2. Has the statistical analysis been performed appropriately and rigorously?

Reviewer #1: Yes

**********

3. Have the authors made all data underlying the findings in their manuscript fully available?

The PLOS Data policy requires authors to make all data underlying the findings described in their manuscript fully available without restriction, with rare exception (please refer to the Data Availability Statement in the manuscript PDF file). The data should be provided as part of the manuscript or its supporting information, or deposited to a public repository. For example, in addition to summary statistics, the data points behind means, medians and variance measures should be available. If there are restrictions on publicly sharing data—e.g. participant privacy or use of data from a third party—those must be specified.

Reviewer #1: Yes

**********

4. Is the manuscript presented in an intelligible fashion and written in standard English?

PLOS ONE does not copyedit accepted manuscripts, so the language in submitted articles must be clear, correct, and unambiguous. Any typographical or grammatical errors should be corrected at revision, so please note any specific errors here.

Reviewer #1: Yes

**********

5. Review Comments to the Author

Please use the space provided to explain your answers to the questions above. You may also include additional comments for the author, including concerns about dual publication, research ethics, or publication ethics. (Please upload your review as an attachment if it exceeds 20,000 characters)

Reviewer #1: the manuscrippt is well designed and novel and the readers shuld be interested in their findings in IUGR deseases.

in fact this method applied to rats may be used futher to hhumans later, in order to reduce liver deseases

**********

6. PLOS authors have the option to publish the peer review history of their article (what does this mean?). If published, this will include your full peer review and any attached files.

If you choose “no”, your identity will remain anonymous but your review may still be made public.

**Do you want your identity to be public for this peer review?** For information about this choice, including consent withdrawal, please see our Privacy Policy.

Reviewer #1: **Yes:** erich cosmi

**********

[NOTE: If reviewer comments were submitted as an attachment file, they will be attached to this email and accessible via the submission site. Please log into your account, locate the manuscript record, and check for the action link "View Attachments". If this link does not appear, there are no attachment files.]

While revising your submission, please upload your figure files to the Preflight Analysis and Conversion Engine (PACE) digital diagnostic tool, https://pacev2.apexcovantage.com/. PACE helps ensure that figures meet PLOS requirements. To use PACE, you must first register as a user. Registration is free. Then, login and navigate to the UPLOAD tab, where you will find detailed instructions on how to use the tool. If you encounter any issues or have any questions when using PACE, please email PLOS at figures@plos.org. Please note that Supporting Information files do not need this step.

# Author comment: Author response to Decision Letter 1

**DOI:** 10.1371/journal.pone.0350859.r002

11 Nov 2025

Comment 1:

Please ensure that your manuscript meets PLOS ONE's style requirements, including those for file naming.

Response 1:

We thank the editor for this reminder. We have carefully reviewed the PLOS ONE style requirements and have revised the manuscript and associated files accordingly. File names have been updated to conform to PLOS ONE’s guidelines.

Comment 2:

To comply with PLOS One submissions requirements, in your Methods section, please provide additional information regarding the experiments involving animals and ensure you have included details on methods of anesthesia and/or analgesia, and efforts to alleviate suffering.

Response 2:

We appreciated the editor’s comment. In the revised methods section, we have added detailed information regarding animal welfare, including the methods of anesthesia and euthanasia. Specifically, we now describe that animals were anesthetized with 1% pentobarbital sodium solution (50 mg/kg, intraperitoneal injection) before procedures, and euthanasia was performed using carbon dioxide inhalation following anesthesia. All experiments were conducted in accordance with institutional ethical guidelines and approved protocols.

Comment 3:

Please include this amended Role of Funder statement in your cover letter.

Response 3:

We have added the role of funder statement in our cover letter.

Comment 4:

Please remove any funding-related text from the manuscript and let us know how you would like to update your Funding Statement.

Response 4:

Thank you for pointing this out, we have removed any funding-related text from the acknowledgments section. The funding Statement should be maintained as follows:

Changsha Municipal Bureau of Science and Technology granted the fundings under project number: [kq2007077].

Comment 5:

Please include a separate caption for each figure in your manuscript.

Response 5:

Separate captions have been included for each figure in the manuscript.

Comment 6:

Please include a copy of Table 1, 2, and 3 which you refer to in your text on page 9 and 11.

Response 6:

Thank you very much, all tables referred to have been added to the manuscript.

Comment 7:

Please include captions for your Supporting Information files at the end of your manuscript, and update any in-text citations to match accordingly. Please see our Supporting Information guidelines for more information: http://journals.plos.org/plosone/s/supporting-information.

Response 7:

Thank you very much, this suggestion will be taken into consideration in case of uploading any supporting materials.

Comment 8:

While the introduction effectively outlines the clinical and physiological significance of IUGR, the transition to puerarin as a proposed intervention appears abrupt and insufficiently substantiated. The manuscript would benefit from a clearer mechanistic rationale linking puerarin’s known biological actions such as its effects on peroxisomes, mitochondrial β-oxidation, or SREBP-1c signaling to the specific metabolic disruptions observed in IUGR models.

Response 8:

We have expanded the introduction to provide a clearer mechanistic rationale, incorporating current literature on IUGR pathophysiology, ensuring a smoother and more substantiated transition to puerarin as the proposed intervention. We highlighted that puerarin:

- Suppresses hepatic lipogenesis by activating AMPK and inhibiting SREBP-1c.

- Enhances mitochondrial fatty acid β-oxidation via upregulation of PPARα, thereby improving lipid utilization.

- Promotes cholesterol efflux through the AMPK-PPARγ-LXR-ABCA1 axis.

See page 3, from line 81 of the revised manuscript.

Comment 9:

The experimental design also raises concerns. The number of animals used (n = 6 per group) seems limited for drawing robust conclusions from molecular and physiological data, especially in the absence of a statistical power analysis.

Response 9:

We sincerely thank the editor for raising this important point. We acknowledge that the sample size of six animals per group is modest. The number was chosen based on previous studies using similar IUGR rodent models where n=6 has been considered sufficient to detect meaningful differences in liver fat content, serum lipids, and molecular markers.

- In a rat IUGR model examining hepatic cholesterol metabolism, n=6 per group was employed to detect differences in cholesterol, LDL receptor levels, and related proteins. [Zinkhan, E., Chin, J., Zalla, J. et al. Combination of intrauterine growth restriction and a high-fat diet impairs cholesterol elimination in rats. Pediatr Res 76, 432–440 (2014)]

- A research into curcumin’s effects on insulin resistance and lipid accumulation in IUGR rats also used n=6 per group, yielding meaningful outcomes across metabolic parameters. [Niu Y, He J, Ahmad H, Wang C, Zhong X, Zhang L, et al. Curcumin attenuates insulin resistance and hepatic lipid accumulation in a rat model of intra-uterine growth restriction through insulin signalling pathway and sterol regulatory element binding proteins. Br J Nutr. 2019;122(6):616-24]

The related literatures have been referenced in the materials and methods section found on page 3, from line 115.

Comment 10:

Additionally, the criteria used to define IUGR status are not clearly stated (e.g., birth weight <2 SD), and it is unclear whether sex was accounted for in group allocation, despite its known influence on lipid metabolism.

Response 10:

In the revised manuscript materials and methods section, we have clarified that IUGR was defined as offspring birth weight below the mean minus two standard deviations of the control group, consistent with established rodent IUGR models. We also specify that, although both male and female offspring were delivered, only male pups were included in the postnatal experimental groups to minimize sex-related variability in lipid metabolism (Page 3, from line 113).

Comment 11:

Furthermore, the rationale behind the selected puerarin dosage and its route of administration is lacking. The manuscript does not address whether this dosage has been validated in neonatal or IUGR models, nor whether it achieves bioactive concentrations in liver tissue. The absence of pharmacokinetic context weakens the interpretation of the intervention’s biological plausibility.

Response 11:

We thank the reviewer for raising this important point. In the revised materials and methods section, we have clarified the rationale for both puerarin dosage and the route of administration. The dosage of 50mg/kg/day was selected based on previous rodent studies in neonatal and metabolic disease models, where puerarin at this level exerted significant protective effects on hepatic injury and lipid metabolism. We chose the intraperitoneal route to ensure consistent systemic exposure in neonatal rats, since oral absorption can be variable at this stage of development. Furthermore, pharmacokinetic and tissue distribution studies have demonstrated that systemically administered puerarin reaches the liver at biologically active concentrations. These clarifications have now been incorporated into the revised manuscript (Page 4, from line 120)

Comment 12:

Importantly, although the study presents gene and protein expression data related to lipid metabolism (e.g., SREBP-1c, FAS, ACC), there is a notable lack of functional metabolic data. Key physiological parameters such as hepatic triglyceride or cholesterol content, markers of insulin sensitivity, or liver enzyme activity are not reported. As a result, claims regarding "improved lipid metabolism" remain speculative without direct biochemical or phenotypic support. A stronger connection between molecular findings and functional outcomes is necessary to substantiate the conclusions.

Response 12:

We thank the editor for this insightful observation. While we agree that additional functional assays, such as direct hepatic triglyceride/cholesterol quantification, insulin sensitivity testing, or liver enzyme activity, would further strengthen the translational relevance, we would like to emphasize that our study already integrates molecular, biochemical, and imaging assessments.

In addition to examining the expression of lipid metabolism using PPARα, a key regulator of hepatic fatty acid oxidation, we also assessed serum lipid profiles (TG, TC, HDL, LDL) and MRI-derived parameters of hepatic fat and perfusion (T1 mapping, ADC, D, D*, and perfusion fraction F) at multiple time points. These endpoints provide both mechanistic and functional evidence of altered lipid metabolism in IUGR rats and its partial restoration with puerarin treatment.

We have, however, revised the discussion to explicitly acknowledge the absence of direct hepatic biochemical assays and insulin sensitivity markers as a limitation and highlighted that future studies will address these aspects to further validate the molecular and functional findings. (Page 12, line 431)

Comment 13:

In terms of language and style, the manuscript would benefit from a more precise and scientifically rigorous tone. Terms such as "improve lipid metabolism" and "protective effect" are overly general and should be clearly defined and supported by specific outcomes. Several statements in the results section also appear speculative, lacking sufficient data backing (e.g., interpretations of PPARα involvement).

Response 13:

Thank you very much for this suggestion, the manuscript language has been revised, and terms have been readjusted to be more precise. And data on PPARα has been indicated in the manuscript.

Comment 14:

Finally, methodological transparency must be improved. The manuscript lacks essential details, such as the complete list of primers used for RT-qPCR, normalization methods for protein expression, and raw numerical data (including means ± SD) underlying the graphs. These omissions hinder reproducibility and limit the reader’s ability to critically assess the findings.

Response 14:

The complete list of of primers used for RT-qPCR, normalization methods for protein expression, and raw numerical data (including means ± SD) underlying the graphs have been added to the manuscript.

Comment 15:

We note that your Data Availability Statement is currently as follows: [All relevant data are within the manuscript.]. Please confirm at this time whether or not your submission contains all raw data required to replicate the results of your study. Authors must share the “minimal data set” for their submission. PLOS defines the minimal data set to consist of the data required to replicate all study findings reported in the article, as well as related metadata and methods (https://journals.plos.org/plosone/s/data-availability#loc-minimal-data-set-definition).

Response 15:

All relevant data supporting the findings of this study are provided within the manuscript and the supplementary materials show extra information on analysis parameters that have been used. In accordance with standards in this field, raw data collected during the investigation are not required for submission; instead, processed data that allow replication and verification of the results are included.

Comment 16:

Thank you for uploading this authorship change form. However, the contributions you have indicated for the authors you are requesting to add would not qualify them for authorship. If this was not in error and the individuals in question have not contributed to all of the three points just mentioned but you would like to recognize these individuals for their contribution to the paper, we recommend including them in the Acknowledgments section of your paper.

Response 16:

Thank you very much for guidance on this matter, we have judged well to recognize the individual in the acknowledgments section after her approval. The acknowledgement section has been modified and written as:

We acknowledge the support of the staff of Central South University's animal facility and laboratory in animal care and experimental procedures.

We also extend our heartfelt gratitude to Doctor Zhou Jun Fang, colleagues and friends who have provided their unwavering assistance for the completion of this research. Be blessed.

Attachment Submitted filename: Response to Reviewers.docx

# Peer review: Decision Letter 1

**DOI:** 10.1371/journal.pone.0350859.r003

**Authors:** Ewa Tomaszewska

21 Dec 2025

PONE-D-25-33662R1

Unraveling Puerarin’s impact on MRI hepatic lipid deposition and serum lipids in IUGR offspring rats

PLOS One

Dear Dr. Mutamba,

Thank you for submitting your manuscript to PLOS ONE. After careful consideration, we feel that it has merit but does not fully meet PLOS ONE’s publication criteria as it currently stands. Therefore, we invite you to submit a revised version of the manuscript that addresses the points raised during the review process.

Please submit your revised manuscript by Feb 04 2026 11:59PM. If you will need more time than this to complete your revisions, please reply to this message or contact the journal office at plosone@plos.org. When you're ready to submit your revision, log on to https://www.editorialmanager.com/pone/ and select the 'Submissions Needing Revision' folder to locate your manuscript file.

Please include the following items when submitting your revised manuscript:

- A letter that responds to each point raised by the academic editor and reviewer(s). You should upload this letter as a separate file labeled 'Response to Reviewers'.
- A marked-up copy of your manuscript that highlights changes made to the original version. You should upload this as a separate file labeled 'Revised Manuscript with Track Changes'.
- An unmarked version of your revised paper without tracked changes. You should upload this as a separate file labeled 'Manuscript'.

If you would like to make changes to your financial disclosure, please include your updated statement in your cover letter. Guidelines for resubmitting your figure files are available below the reviewer comments at the end of this letter.

If applicable, we recommend that you deposit your laboratory protocols in protocols.io to enhance the reproducibility of your results. Protocols.io assigns your protocol its own identifier (DOI) so that it can be cited independently in the future. For instructions see: https://journals.plos.org/plosone/s/submission-guidelines#loc-laboratory-protocols. Additionally, PLOS ONE offers an option for publishing peer-reviewed Lab Protocol articles, which describe protocols hosted on protocols.io. Read more information on sharing protocols at https://plos.org/protocols?utm_medium=editorial-email&utm_source=authorletters&utm_campaign=protocols.

We look forward to receiving your revised manuscript.

Kind regards,

Ewa Tomaszewska, DVM Ph.D

Academic Editor

PLOS One

Journal Requirements:

If the reviewer comments include a recommendation to cite specific previously published works, please review and evaluate these publications to determine whether they are relevant and should be cited. There is no requirement to cite these works unless the editor has indicated otherwise.

[Note: HTML markup is below. Please do not edit.]

Reviewers' comments:

Reviewer's Responses to Questions

**Comments to the Author**

1. If the authors have adequately addressed your comments raised in a previous round of review and you feel that this manuscript is now acceptable for publication, you may indicate that here to bypass the “Comments to the Author” section, enter your conflict of interest statement in the “Confidential to Editor” section, and submit your "Accept" recommendation.

Reviewer #2: All comments have been addressed

Reviewer #3: (No Response)

**********

2. Is the manuscript technically sound, and do the data support the conclusions?

The manuscript must describe a technically sound piece of scientific research with data that supports the conclusions. Experiments must have been conducted rigorously, with appropriate controls, replication, and sample sizes. The conclusions must be drawn appropriately based on the data presented.

Reviewer #2: Partly

Reviewer #3: Partly

**********

3. Has the statistical analysis been performed appropriately and rigorously?

Reviewer #2: Yes

Reviewer #3: No

**********

4. Have the authors made all data underlying the findings in their manuscript fully available?

The PLOS Data policy requires authors to make all data underlying the findings described in their manuscript fully available without restriction, with rare exception (please refer to the Data Availability Statement in the manuscript PDF file). The data should be provided as part of the manuscript or its supporting information, or deposited to a public repository. For example, in addition to summary statistics, the data points behind means, medians and variance measures should be available. If there are restrictions on publicly sharing data—e.g. participant privacy or use of data from a third party—those must be specified.

Reviewer #2: Yes

Reviewer #3: Yes

**********

5. Is the manuscript presented in an intelligible fashion and written in standard English?

PLOS ONE does not copyedit accepted manuscripts, so the language in submitted articles must be clear, correct, and unambiguous. Any typographical or grammatical errors should be corrected at revision, so please note any specific errors here.

Reviewer #2: (No Response)

Reviewer #3: Yes

**********

6. Review Comments to the Author

Please use the space provided to explain your answers to the questions above. You may also include additional comments for the author, including concerns about dual publication, research ethics, or publication ethics. (Please upload your review as an attachment if it exceeds 20,000 characters)

Reviewer #2: Comment:

1. Please make sure all the figures should be clear

2. ANOVA description is incomplete OR incorrect, it state that statistical analysis was performed using one-way ANOVA but the study has 3 groups (Control, IUGR, IUGR + puerarin) and 3 time points (weeks 3, 8, 12). A one-way ANOVA is not appropriate because the design involves two factors: Treatment group and Time Correct approach should be: Two-way ANOVA, or Repeated-measures ANOVA, or Mixed-model ANOVA. Incorrect statistical method reported., please justify or correct it.

3. Conflicting MRI results at week 3: significant differences between control and IUGR and IUGR plus puerarin (week 3) but puerarin treatment starts after birth, so: At week 3, the puerarin group should already differ from IUGR (if treatment works). Yet the MRI shows puerarin still identical to IUGR. This contradicts the conclusion that puerarin shows early-life improvement. This is either: A biological inconsistency, or Reporting error please clarify.

4. Incomplete reporting of MRI parameters MRI parameters (T1, D, ADC, F, D*) are mentioned, but: Direction of change is never specified (↑ or ↓). No effect sizes or biological interpretation. Only weeks 3, 8, 12 differences are listed. Without describing the direction of abnormalities, the results section is incomplete and unclear.

5. The reported IVIM/ADC values in Table 3 are inconsistent with standard units and magnitudes for biological diffusion (values reported as D ≈ 1271 mm²/s and ADC ≈ 1562 mm²/s). Please correct the units and/or scaling (likely ×10⁻³ or ×10⁻⁶) and provide an example calculation or raw output to verify values

6. Outdated references used, I recommend adding more recent literature (past 5 years).

7. Lack of direct hepatic lipid quantification / functional endpoints. The study relies on MRI and serum lipids but does not report direct hepatic triglyceride/cholesterol assays, liver enzymes, or insulin sensitivity measures — the authors acknowledge this but still make claims about “improved lipid metabolism.” Strongly recommend adding if not, require language in the discussion that conclusions are provisional.

8. Incorrect or inconsistent reference formatting

9. The manuscript contains numerous grammatical errors, inconsistent terminology and unclear or repetitive sentences that hinder readability. Please check Grammarly

Reviewer #3: General comments

Thank you for the privilege to review your research paper. I acknowledge the amount of effort that went into the investigation. The methodology that is applied appears elegant but the scientific integrity, specifically of the method section is lacking.

Specific comments

Abstract

Most previous comments have been addressed, but are not carried through to the abstract, e.g. the abrupt transition to puerarin. The introductory paragraph of the abstract should conclude with the rationale for using puerarin specifically as a therapeutic agent in this study. State the dosage, route of administration, and week of commencement of treatment in the methods component of the abstract.

‘Whereas puerarin intervention notably reduced serum TG, TC, and LDL levels in IUGR rats.’ Needs to be rephrased – it is not a full sentence.

Use abbreviations only if a term appears multiple times within the abstract.

Manuscript body

Statistical analysis: The standard approach for a study with such a small sample size is to use non-parametric tests (after evaluating the distribution), in particular the Kruskal-Wallis test, using the Conover or Dunn’s test for pairwise comparisons. Please consult a statistician.

Bar graphs of parameters are not ideal because the distribution of the data points is poorly visualized. Box-and-Whisker plots or scatter/dot plots, which are connected to the median and range values shown in the tables, are most likely more suitable. Include the units of measurement in the y-axis title. The images depicted in Fig 2:P Liver T1 mapping and IVIM MRI, sadly, are of poor quality. The script is intelligible and the aspect ratios of the 2nd and 3rd column images are distorted. Please consider improving them.

Puerarin administration comment: Puerarin has low water solubility, as you may have noticed. Both puerarin and the 5%D/W solution are irritants and could cause harm and pain when administered intraperitoneally and thereafter due to inflammation. A frequently cited formulation is 10% DMSO + 40% PEG300 + 5% Tween-80 + 45% Saline, as is a 0,9% NaCl solution. (For future reference)

Lack of reference to clinical (human) relevance and the feasibility of intervention

The statement that ‘Early-life interventions that target PPARα-mediated lipid regulation and liver function may represent promising avenues to improve lifelong health’ is rather presumptuous, considering the current status of the research in humans. The current data suggest potential benefits in in vitro and animal models, but significant safety concerns, particularly regarding development, and a complete absence of human neonatal clinical trial data mean it is not an approved or recommended treatment for this population. Consider ‘softening’ the statement.

Best wishes for your future research!

**********

7. PLOS authors have the option to publish the peer review history of their article (what does this mean?). If published, this will include your full peer review and any attached files.

If you choose “no”, your identity will remain anonymous but your review may still be made public.

**Do you want your identity to be public for this peer review?** For information about this choice, including consent withdrawal, please see our Privacy Policy.

Reviewer #2: No

Reviewer #3: No

**********

[NOTE: If reviewer comments were submitted as an attachment file, they will be attached to this email and accessible via the submission site. Please log into your account, locate the manuscript record, and check for the action link "View Attachments". If this link does not appear, there are no attachment files.]

To ensure your figures meet our technical requirements, please review our figure guidelines: https://journals.plos.org/plosone/s/figures

You may also use PLOS’s free figure tool, NAAS, to help you prepare publication quality figures: https://journals.plos.org/plosone/s/figures#loc-tools-for-figure-preparation.

NAAS will assess whether your figures meet our technical requirements by comparing each figure against our figure specifications.

# Author comment: Author response to Decision Letter 2

**DOI:** 10.1371/journal.pone.0350859.r004

26 Feb 2026

Reviewer 2

Comment 1:

Please make sure all the figures should be clear.

Response 1:

We thank the reviewer for this important suggestion. We have revised all figures to ensure clarity.

Comment 2:

ANOVA description is incomplete OR incorrect, it state that statistical analysis was performed using one-way ANOVA but the study has 3 groups (Control, IUGR, IUGR + puerarin) and 3 time points (weeks 3, 8, 12). A one-way ANOVA is not appropriate because the design involves two factors: Treatment group and Time Correct approach should be: Two-way ANOVA, or Repeated-measures ANOVA, or Mixed-model ANOVA. Incorrect statistical method reported., please justify or correct it.

Response 2:

We thank the reviewer for this insightful comment and for identifying this error in our statistical reporting. We agree that a one-way ANOVA was not the appropriate method for this experimental design, which involves two independent factors: Treatment Group and Time. We have corrected the 'Statistical Analysis' section of the manuscript to state that a Two-way ANOVA was utilized. This approach allowed us to assess the main effects of the treatment, the effect of time, and the interaction between these two variables. Multiple comparisons were performed using Tukey’s post hoc test to determine significance between groups at individual time points. The revised methodology now accurately reflects the statistical approach used to analyze the study data, and the results have been verified accordingly.

Comment 3:

Conflicting MRI results at week 3: significant differences between control and IUGR and IUGR plus puerarin (week 3) but puerarin treatment starts after birth, so: At week 3, the puerarin group should already differ from IUGR (if treatment works). Yet the MRI shows puerarin still identical to IUGR. This contradicts the conclusion that puerarin shows early-life improvement. This is either: A biological inconsistency, or Reporting error please clarify.

Response 3:

We appreciate the reviewer’s rigorous evaluation of the Week 3 data. We would like to clarify that while puerarin administration commenced immediately postnatal, the absence of a significant difference between the IUGR and IUGR plus puerarin groups at the Week 3 MRI timepoint does not indicate a failure of the treatment, but rather reflects the temporal latency required for pharmacological intervention to reverse established intrauterine structural or metabolic programming.

Intrauterine Growth Restriction (IUGR) induces profound developmental adaptations in utero. Although puerarin treatment was initiated shortly after birth, the three-week interval may have been insufficient to manifest detectable changes via MRI, despite the treatment being active. The "early-life improvement" we refer to is characterized by the divergence observed starting between Week 3 and Week 8, where the puerarin group begins to shift toward the Control profile while the untreated IUGR group progresses toward a more severe dyslipidemic state. We have revised the Discussion to more accurately characterize Week 3 as the "initial post-intervention baseline" and clarified that the therapeutic efficacy of puerarin follows a cumulative trajectory that becomes statistically evident by Week 8 and Week 12.

Comment 4:

Incomplete reporting of MRI parameters MRI parameters (T1, D, ADC, F, D*) are mentioned, but: Direction of change is never specified (↑ or ↓). No effect sizes or biological interpretation. Only weeks 3, 8, 12 differences are listed. Without describing the direction of abnormalities, the results section is incomplete and unclear.

Response 4:

We thank the reviewer for this constructive critique. We agree that specifying the direction of change and providing a biological interpretation is essential for a complete understanding of the MRI data.

We have revised the Results section to explicitly state the direction of change for each parameter (T1, D, ADC, f, and D*). Furthermore, we have expanded the Discussion to include the biological implications of these findings—specifically how alterations in water diffusion (D, ADC) and perfusion fractions (f, D*) reflect the progressive hepatic or metabolic impairment in the IUGR model and the subsequent restorative effects of puerarin.

Comment 5:

The reported IVIM/ADC values in Table 3 are inconsistent with standard units and magnitudes for biological diffusion (values reported as D ≈ 1271 mm²/s and ADC ≈ 1562 mm²/s). Please correct the units and/or scaling (likely ×10⁻³ or ×10⁻⁶) and provide an example calculation or raw output to verify values.

Response 5:

Thank you very much for pointing this out. We have corrected the MRI parameters Table across all time points (Weeks 3, 8, and 12). These corrected magnitudes now align with established physiological ranges for hepatic tissue.

Comment 6:

Outdated references used, I recommend adding more recent literature (past 5 years).

Response 6:

We have added more recent references from past 5 years, thank you very much.

Comment 7:

Lack of direct hepatic lipid quantification / functional endpoints. The study relies on MRI and serum lipids but does not report direct hepatic triglyceride/cholesterol assays, liver enzymes, or insulin sensitivity measures — the authors acknowledge this but still make claims about “improved lipid metabolism.” Strongly recommend adding if not, require language in the discussion that conclusions are provisional.

Response 7:

Thank you very much for this comment, we have accordingly adjusted the language of the manuscript.

Comment 8:

Incorrect or inconsistent reference formatting.

Response 8:

We apologize for the inconsistencies in the reference list. We have conducted a thorough review of the bibliography. Thank you very much.

Comment 9:

The manuscript contains numerous grammatical errors, inconsistent terminology and unclear or repetitive sentences that hinder readability. Please check Grammarly.

Response 9:

We sincerely apologize for the linguistic oversights in the original submission. We recognize that clear communication is vital for the dissemination of scientific findings. In response to your feedback, the entire manuscript has undergone a comprehensive revision for clarity, grammar, and style.

Reviewer 3

Comment 1:

Most previous comments have been addressed, but are not carried through to the abstract, e.g. the abrupt transition to puerarin. The introductory paragraph of the abstract should conclude with the rationale for using puerarin specifically as a therapeutic agent in this study. State the dosage, route of administration, and week of commencement of treatment in the methods component of the abstract.

Response 1:

We thank the reviewer for pointing out these omissions in the abstract. We have fully revised the abstract to ensure consistency with the main text. Specifically: We added a concluding sentence to the introductory section explaining the choice of puerarin based on its known antioxidant and lipid-modulating properties. We now explicitly state the dosage (50 mg/kg/d), the route of administration (intraperitoneal injection), and the treatment window (postnatal days 7 to 21). We have adjusted the transition to puerarin to be more fluid and logically connected to the study's aim of restoring PPARα activity.

Comment 2:

‘Whereas puerarin intervention notably reduced serum TG, TC, and LDL levels in IUGR rats.’ Needs to be rephrased – it is not a full sentence.

Response 2:

We apologize for the grammatical error. We have rephrased this statement to ensure it is a complete, grammatically correct sentence that clearly describes the therapeutic effect observed.

Comment 3:

Use abbreviations only if a term appears multiple times within the abstract.

Response 3:

We thank the reviewer for this suggestion. We have reviewed the abstract and ensured that all abbreviations are defined at their first usage and subsequently used consistently throughout the text.

Comment 4:

Statistical analysis: The standard approach for a study with such a small sample size is to use non-parametric tests (after evaluating the distribution), in particular the Kruskal-Wallis test, using the Conover or Dunn’s test for pairwise comparisons. Please consult a statistician.

Bar graphs of parameters are not ideal because the distribution of the data points is poorly visualized. Box-and-Whisker plots or scatter/dot plots, which are connected to the median and range values shown in the tables, are most likely more suitable. Include the units of measurement in the y-axis title. The images depicted in Fig 2:P Liver T1 mapping and IVIM MRI, sadly, are of poor quality. The script is intelligible and the aspect ratios of the 2nd and 3rd column images are distorted. Please consider improving them.

Response 4:

We thank the reviewer for the suggestion to employ nonparametric testing given our sample size (n=6). We have updated Figures and Tables to present data as Median [Interquartile Range]. Furthermore, inter-group comparisons at each timepoint were recalculated using Kruskal-Wallis tests followed by Dunn’s post-hoc criteria. While this conservative approach slightly adjusted some p-values, the overall longitudinal trends remain robust.

Comment 5:

Puerarin administration comment: Puerarin has low water solubility, as you may have noticed. Both puerarin and the 5%D/W solution are irritants and could cause harm and pain when administered intraperitoneally and thereafter due to inflammation. A frequently cited formulation is 10% DMSO + 40% PEG300 + 5% Tween-80 + 45% Saline, as is a 0,9% NaCl solution. (For future reference).

Response 5:

We thank the reviewer for this insightful technical comment regarding the solubility and potential irritant properties of the puerarin formulation. We acknowledge that puerarin's low water solubility often necessitates specialized vehicles like DMSO or PEG for optimal delivery. In this study, we utilized a 5% glucose (D/W) solution as a vehicle based on prior established protocols for neonatal rat models, aiming to minimize the use of organic solvents like DMSO, which can sometimes exert independent metabolic or neuroprotective effects in developing pups. Throughout the 14-day administration period (P7–P21), all pups were closely monitored for signs of abdominal tenderness, lethargy, or behavioral changes. No adverse reactions or significant signs of distress were observed. During the scheduled sacrifices at weeks 3, 8, and 12, the peritoneal cavity and liver surface were inspected; no gross evidence of peritonitis, adhesions, or chronic inflammation was identified. We appreciate the reviewer’s suggestion of the DMSO/PEG300/Tween-80 formulation and will certainly adopt this more robust vehicle for our future longitudinal studies to ensure optimal solubility and minimize tissue irritation.

Comment 6:

Lack of reference to clinical (human) relevance and the feasibility of intervention.

Response 6:

We appreciate the reviewer highlighting the need to contextualize our findings within a clinical framework. We have expanded our Discussion to explicitly address the clinical relevance of this study and the translation of puerarin intervention.

Comment 7:

The statement that ‘Early-life interventions that target PPARα-mediated lipid regulation and liver function may represent promising avenues to improve lifelong health’ is rather presumptuous, considering the current status of the research in humans. The current data suggt potential benefits in in vitro and animal models, but significant safety concerns, particularly regarding development, and a complete absence of human neonatal clinical trial data mean it is not an approved or recommended treatment for this population. Consider ‘softening’ the statement.

Response 7:

We completely agree with the reviewer’s assessment and thank them for this important correction. Our intention was to highlight the biological potential discovered in our model, but we recognize that the original phrasing overstated the clinical readiness of this intervention. We have "softened" the language throughout the manuscript, particularly in the Abstract and Conclusion. We now explicitly state that these findings are limited to a preclinical rat model and that puerarin is currently neither an approved nor a recommended treatment for human neonates. We have also added a section in the Discussion addressing the significant safety and developmental hurdles that must be cleared before any human neonatal trials could be considered.

Attachment Submitted filename: Response_to_Reviewers_auresp_2.docx

# Peer review: Decision Letter 2

**DOI:** 10.1371/journal.pone.0350859.r005

**Authors:** Ewa Tomaszewska

23 Mar 2026

PONE-D-25-33662R2

Unraveling Puerarin’s impact on MRI hepatic lipid deposition and serum lipids in IUGR offspring rats

PLOS One

Dear Dr. Mutamba,

Thank you for submitting your manuscript to PLOS ONE. After careful consideration, we feel that it has merit but does not fully meet PLOS ONE’s publication criteria as it currently stands. Therefore, we invite you to submit a revised version of the manuscript that addresses the points raised during the review process.

Please submit your revised manuscript by May 07 2026 11:59PM. If you will need more time than this to complete your revisions, please reply to this message or contact the journal office at plosone@plos.org. When you're ready to submit your revision, log on to https://www.editorialmanager.com/pone/ and select the 'Submissions Needing Revision' folder to locate your manuscript file.

Please include the following items when submitting your revised manuscript:

- A letter that responds to each point raised by the academic editor and reviewer(s). You should upload this letter as a separate file labeled 'Response to Reviewers'.
- A marked-up copy of your manuscript that highlights changes made to the original version. You should upload this as a separate file labeled 'Revised Manuscript with Track Changes'.
- An unmarked version of your revised paper without tracked changes. You should upload this as a separate file labeled 'Manuscript'.

If you would like to make changes to your financial disclosure, please include your updated statement in your cover letter. Guidelines for resubmitting your figure files are available below the reviewer comments at the end of this letter.

If applicable, we recommend that you deposit your laboratory protocols in protocols.io to enhance the reproducibility of your results. Protocols.io assigns your protocol its own identifier (DOI) so that it can be cited independently in the future. For instructions see: https://journals.plos.org/plosone/s/submission-guidelines#loc-laboratory-protocols. Additionally, PLOS ONE offers an option for publishing peer-reviewed Lab Protocol articles, which describe protocols hosted on protocols.io. Read more information on sharing protocols at https://plos.org/protocols?utm_medium=editorial-email&utm_source=authorletters&utm_campaign=protocols.

We look forward to receiving your revised manuscript.

Kind regards,

Ewa Tomaszewska, DVM Ph.D

Academic Editor

PLOS One

Journal Requirements:

If the reviewer comments include a recommendation to cite specific previously published works, please review and evaluate these publications to determine whether they are relevant and should be cited. There is no requirement to cite these works unless the editor has indicated otherwise.

[Note: HTML markup is below. Please do not edit.]

Reviewers' comments:

Reviewer's Responses to Questions

**Comments to the Author**

1. If the authors have adequately addressed your comments raised in a previous round of review and you feel that this manuscript is now acceptable for publication, you may indicate that here to bypass the “Comments to the Author” section, enter your conflict of interest statement in the “Confidential to Editor” section, and submit your "Accept" recommendation.

Reviewer #2: (No Response)

Reviewer #3: (No Response)

**********

2. Is the manuscript technically sound, and do the data support the conclusions?

The manuscript must describe a technically sound piece of scientific research with data that supports the conclusions. Experiments must have been conducted rigorously, with appropriate controls, replication, and sample sizes. The conclusions must be drawn appropriately based on the data presented.

Reviewer #2: Yes

Reviewer #3: No

**********

3. Has the statistical analysis been performed appropriately and rigorously?

Reviewer #2: Yes

Reviewer #3: No

**********

4. Have the authors made all data underlying the findings in their manuscript fully available?

The PLOS Data policy requires authors to make all data underlying the findings described in their manuscript fully available without restriction, with rare exception (please refer to the Data Availability Statement in the manuscript PDF file). The data should be provided as part of the manuscript or its supporting information, or deposited to a public repository. For example, in addition to summary statistics, the data points behind means, medians and variance measures should be available. If there are restrictions on publicly sharing data—e.g. participant privacy or use of data from a third party—those must be specified.

Reviewer #2: (No Response)

Reviewer #3: Yes

**********

5. Is the manuscript presented in an intelligible fashion and written in standard English?

PLOS ONE does not copyedit accepted manuscripts, so the language in submitted articles must be clear, correct, and unambiguous. Any typographical or grammatical errors should be corrected at revision, so please note any specific errors here.

Reviewer #2: Yes

Reviewer #3: Yes

**********

6. Review Comments to the Author

Please use the space provided to explain your answers to the questions above. You may also include additional comments for the author, including concerns about dual publication, research ethics, or publication ethics. (Please upload your review as an attachment if it exceeds 20,000 characters)

Reviewer #2: The manuscript investigates the potential protective effects of puerarin on liver alterations associated with intrauterine growth restriction (IUGR) using MRI parameters, biochemical measurements, and gene expression analysis. The topic is interesting and potentially relevant to understanding metabolic programming in early life. However, several methodological, statistical, and interpretational issues limit the reliability of the conclusions. Substantial clarification and additional analysis are required before the manuscript can be considered for publication.

1. MRI data is collected longitudinally (weeks 3, 8, 12), but analyses assume independent observations. If the same animals were followed, appropriate methods (repeated-measures ANOVA or mix-effects models) should be used. Current analysis may yield unreliable results.

2. T1 mapping and IVIM diffusion are non-specific and do not directly measure hepatic fat. Conclusions about lipid deposition are not adequately supported without validation (e.g., PDFF, MR spectroscopy, or histology).

3. Reported diffusion/ADC values appear far below expected physiological ranges, suggesting possible unit or scaling errors. These should be verified and corrected.

4. The manuscript attributes effects to PPARα activation despite non-significant changes in expression. This represents overinterpretation and should be revised or better supported.

5. Effects at week 12 are attributed to early treatment (weeks 1–3) without controlling for confounders (e.g., growth, diet), limiting causal interpretation.

6. The manuscript requires language and grammar editing for clarity.

7. Ensure consistent terminology and definition of abbreviations.

8. Several claims require stronger and more appropriate references, particularly regarding MRI-based lipid assessment.

9. Add methodological details for MRI analysis (e.g., b-values, ROI selection, blinding).

10. Verify reference formatting and completeness.

Reviewer #3: The proposed analysis is problematic because it switches between parametric (ANOVA) and non-parametric (Kruskal-Wallis) tests for the same data; you should stick to one statistical framework.

Please consult a statistician

**********

7. PLOS authors have the option to publish the peer review history of their article (what does this mean?). If published, this will include your full peer review and any attached files.

If you choose “no”, your identity will remain anonymous but your review may still be made public.

**Do you want your identity to be public for this peer review?** For information about this choice, including consent withdrawal, please see our Privacy Policy.

Reviewer #2: No

Reviewer #3: No

**********

[NOTE: If reviewer comments were submitted as an attachment file, they will be attached to this email and accessible via the submission site. Please log into your account, locate the manuscript record, and check for the action link "View Attachments". If this link does not appear, there are no attachment files.]

To ensure your figures meet our technical requirements, please review our figure guidelines: https://journals.plos.org/plosone/s/figures

You may also use PLOS’s free figure tool, NAAS, to help you prepare publication quality figures: https://journals.plos.org/plosone/s/figures#loc-tools-for-figure-preparation.

NAAS will assess whether your figures meet our technical requirements by comparing each figure against our figure specifications.

# Author comment: Author response to Decision Letter 3

**DOI:** 10.1371/journal.pone.0350859.r006

6 May 2026

Response to Reviewers

Manuscript title: Unraveling Puerarin’s impact on MRI hepatic lipid deposition and serum lipids in IUGR offspring rats

Manuscript ID: PONE-D-25-33662R2

Dear Academic Editor and Reviewers,

We thank you very much for your constructive feedback on our manuscript. We have carefully revised the paper in response to your suggestions. Below, we have provide a point-by-point response to each comment, and all changes have been highlighted in the revised manuscript.

Reviewer 2

Comment 1:

MRI data is collected longitudinally (weeks 3, 8, 12), but analyses assume independent observations. If the same animals were followed, appropriate methods (repeated-measures ANOVA or mix-effects models) should be used. Current analysis may yield unreliable results.

Response 1:

We thank the reviewer for this comment and apologize for the lack of clarity regarding our study design. While the data represents different time points (Weeks 3, 8, and 12), these measurements were conducted on separate cohorts of rats at each interval rather than following the same rats. Consequently, the observations are independent, and the use of Two-Way ANOVA is statistically appropriate. We have clarified this in the revised manuscript and updated the figure legends to reflect that each time point represents a distinct group.

Comment 2:

T1 mapping and IVIM diffusion are non-specific and do not directly measure hepatic fat. Conclusions about lipid deposition are not adequately supported without validation (e.g., PDFF, MR spectroscopy, or histology).

Response 2:

We appreciate this important methodological clarification. We acknowledge that T1 mapping and IVIM-derived parameters are indirect, non-specific biomarkers and do not directly quantify hepatic fat content. In the revised manuscript, we have carefully removed all statements implying direct measurement of lipid deposition and replaced them with appropriately cautious terminology referring to hepatic microstructural alterations and water diffusion changes.

Comment 3:

Reported diffusion/ADC values appear far below expected physiological ranges, suggesting possible unit or scaling errors. These should be verified and corrected.

Response 3:

We thank the reviewer for identifying this important issue.In the revised manuscript, we have standardized all diffusion-related parameters and clarified that ADC and D values are reported in units of ×10⁻³ mm²/s.

Comment 4:

The manuscript attributes effects to PPARα activation despite non-significant changes in expression. This represents overinterpretation and should be revised or better supported.

Response 4:

We appreciate this important critique. We acknowledge that our previous interpretation overemphasized the mechanistic role of PPARα. In the revised manuscript, we have tempered all causal statements and clarified that although puerarin significantly increased PPARα expression at week 12, expression remained below control levels. We now interpret PPARα changes as a contributing but not exclusive mechanism, and we have incorporated additional discussion of alternative pathways including AMPK/SREBP-1c signaling and anti-inflammatory effects.

Comment 5:

Effects at week 12 are attributed to early treatment (weeks 1–3) without controlling for confounders (e.g., growth, diet), limiting causal interpretation.

Response 5:

We agree with the reviewer that causal interpretation of long-term effects is limited by potential confounding factors such as growth and developmental progression.We now clearly state that while improvements observed at week 12 coincide with early puerarin administration, definitive causal inference cannot be established within the current study design.

Comment 6:

The manuscript requires language and grammar editing for clarity.

Response 6:

We thank the reviewer for this suggestion. The manuscript has undergone comprehensive language editing and grammatical revision to improve clarity, conciseness, and scientific readability.

Comment 7:

Ensure consistent terminology and definition of abbreviations.

Response 7:

We appreciate this comment.All abbreviations have now been defined upon first use and applied uniformly across text, tables, and figures, including IVIM-derived parameters (D, D*, F), lipid profiles (TG, TC, HDL-C, LDL-C), and PPARα nomenclature.

Comment 8:

Several claims require stronger and more appropriate references, particularly regarding MRI-based lipid assessment.

Response 8:

We thank the reviewer for this valuable suggestion.We have clarified the limitations of MRI in directly quantifying hepatic fat in the absence of PDFF or MR spectroscopy.

Comment 9:

Add methodological details for MRI analysis (e.g., b-values, ROI selection, blinding).

Response 9:

We thank the reviewer for identifying these omissions. We have significantly expanded the MRI Acquisition and Analysis section to include these critical details. Specifically:b-values: We have explicitly listed the nine b-values used for IVIM (0, 25, 50, 100, 150, 300, 500, 800, 1000s/mm2). About ROI Selection: We have detailed the size (11–26 mm2 for T1 mapping; 50 mm2 for IVIM) and the placement (liver parenchyma, avoiding large vessels) of the regions of interest. For blinding: We have clarified that all image post-processing and parameter extraction were performed by a radiologist blinded to the experimental group assignments to ensure objectivity.

Comment 10:

Verify reference formatting and completeness.

Response 10:

Thank you very much for this comment, the reference format has been checked.

Reviewer 3

Comment 1:

The proposed analysis is problematic because it switches between parametric (ANOVA) and non-parametric (Kruskal-Wallis) tests for the same data; you should stick to one statistical framework.

Response 1:

We thank the reviewer for this important statistical observation. We agree that using both parametric and non-parametric approaches for the same dataset could create inconsistency in interpretation. In the revised manuscript, we standardized the statistical analysis framework across all continuous variables. Specifically, all outcomes were analyzed using two-way ANOVA (group × time) followed by Tukey’s multiple-comparison post hoc test.

Attachment Submitted filename: Response_to_Reviewers_auresp_3.docx

# Peer review: Decision Letter 3

**DOI:** 10.1371/journal.pone.0350859.r007

**Authors:** Ewa Tomaszewska

19 May 2026

Unraveling Puerarin’s impact on MRI hepatic lipid deposition and serum lipids in IUGR offspring rats

PONE-D-25-33662R3

Dear Dr. Kalonda Alpha Mutamba,

We’re pleased to inform you that your manuscript has been judged scientifically suitable for publication and will be formally accepted for publication once it meets all outstanding technical requirements.

Within one week, you’ll receive an e-mail detailing the required amendments. When these have been addressed, you’ll receive a formal acceptance letter and your manuscript will be scheduled for publication.

An invoice will be generated when your article is formally accepted. Please note, if your institution has a publishing partnership with PLOS and your article meets the relevant criteria, all or part of your publication costs will be covered. Please make sure your user information is up-to-date by logging into Editorial Manager at Editorial Manager® and clicking the ‘Update My Information' link at the top of the page. For questions related to billing, please contact billing support.

If your institution or institutions have a press office, please notify them about your upcoming paper to help maximize its impact. If they’ll be preparing press materials, please inform our press team as soon as possible -- no later than 48 hours after receiving the formal acceptance. Your manuscript will remain under strict press embargo until 2 pm Eastern Time on the date of publication. For more information, please contact onepress@plos.org.

Kind regards,

Ewa Tomaszewska, DVM Ph.D

Academic Editor

PLOS One

Additional Editor Comments (optional):

Reviewers' comments:

Reviewer's Responses to Questions

**Comments to the Author**

1. If the authors have adequately addressed your comments raised in a previous round of review and you feel that this manuscript is now acceptable for publication, you may indicate that here to bypass the “Comments to the Author” section, enter your conflict of interest statement in the “Confidential to Editor” section, and submit your "Accept" recommendation.

Reviewer #2: All comments have been addressed

Reviewer #3: All comments have been addressed

**********

2. Is the manuscript technically sound, and do the data support the conclusions?

The manuscript must describe a technically sound piece of scientific research with data that supports the conclusions. Experiments must have been conducted rigorously, with appropriate controls, replication, and sample sizes. The conclusions must be drawn appropriately based on the data presented.

Reviewer #2: Yes

Reviewer #3: Yes

**********

3. Has the statistical analysis been performed appropriately and rigorously?

Reviewer #2: Yes

Reviewer #3: Yes

**********

4. Have the authors made all data underlying the findings in their manuscript fully available?

The PLOS Data policy requires authors to make all data underlying the findings described in their manuscript fully available without restriction, with rare exception (please refer to the Data Availability Statement in the manuscript PDF file). The data should be provided as part of the manuscript or its supporting information, or deposited to a public repository. For example, in addition to summary statistics, the data points behind means, medians and variance measures should be available. If there are restrictions on publicly sharing data—e.g. participant privacy or use of data from a third party—those must be specified.

Reviewer #2: Yes

Reviewer #3: Yes

**********

5. Is the manuscript presented in an intelligible fashion and written in standard English?

PLOS ONE does not copyedit accepted manuscripts, so the language in submitted articles must be clear, correct, and unambiguous. Any typographical or grammatical errors should be corrected at revision, so please note any specific errors here.

Reviewer #2: Yes

Reviewer #3: Yes

**********

6. Review Comments to the Author

Please use the space provided to explain your answers to the questions above. You may also include additional comments for the author, including concerns about dual publication, research ethics, or publication ethics. (Please upload your review as an attachment if it exceeds 20,000 characters)

Reviewer #2: (No Response)

Reviewer #3: Considerable revision of the manuscript is evident. The "Comments to the Author” section has been bypassed since all concerns were addressed.

**********

7. PLOS authors have the option to publish the peer review history of their article (what does this mean?). If published, this will include your full peer review and any attached files.

If you choose “no”, your identity will remain anonymous but your review may still be made public.

**Do you want your identity to be public for this peer review?** For information about this choice, including consent withdrawal, please see our Privacy Policy.

Reviewer #2: No

Reviewer #3: No

**********

# Editor report: Acceptance letter

**DOI:** 10.1371/journal.pone.0350859.r008

**Authors:** Ewa Tomaszewska

PONE-D-25-33662R3

PLOS One

Dear Dr. Mutamba,

I'm pleased to inform you that your manuscript has been deemed suitable for publication in PLOS One. Congratulations! Your manuscript is now being handed over to our production team.

At this stage, our production department will prepare your paper for publication. This includes ensuring the following:

* All references, tables, and figures are properly cited

* All relevant supporting information is included in the manuscript submission,

* There are no issues that prevent the paper from being properly typeset

You will receive further instructions from the production team, including instructions on how to review your proof when it is ready. Please keep in mind that we are working through a large volume of accepted articles, so please give us a few days to review your paper and let you know the next and final steps.

Lastly, if your institution or institutions have a press office, please let them know about your upcoming paper now to help maximize its impact. If they'll be preparing press materials, please inform our press team within the next 48 hours. Your manuscript will remain under strict press embargo until 2 pm Eastern Time on the date of publication. For more information, please contact onepress@plos.org.

You will receive an invoice from PLOS for your publication fee after your manuscript has reached the completed accept phase. If you receive an email requesting payment before acceptance or for any other service, this may be a phishing scheme. Learn how to identify phishing emails and protect your accounts at https://explore.plos.org/phishing.

If we can help with anything else, please email us at customercare@plos.org.

Thank you for submitting your work to PLOS ONE and supporting open access.

Kind regards,

PLOS ONE Editorial Office Staff

on behalf of

Professor Ewa Tomaszewska

Academic Editor

PLOS One
